# On the trail of Scandinavia’s early metallurgy: Provenance, transfer and mixing

**DOI:** 10.1371/journal.pone.0219574

**Published:** 2019-07-24

**Authors:** Heide W. Nørgaard, Ernst Pernicka, Helle Vandkilde

**Affiliations:** 1 Department of Archaeology and Heritage Studies, School of Culture and Society, Aarhus University, Aarhus-Højbjerg, Denmark; 2 Curt-Engelhorn-Center for Archaeometry, Mannheim, Germany; 3 Institute for Geosciences, Ruprecht-Karls University Heidelberg, Heidelberg, Germany; University at Buffalo - The State University of New York, UNITED STATES

## Abstract

The rich and long-lasting Nordic Bronze Age was dependent throughout on incoming flows of copper and tin. The crucial turning point for the development of the NBA can be pinpointed as the second phase of the Late Neolithic (LN II, c. 2000–1700 BC) precisely because the availability and use of metal increased markedly at this time. But the precise provenance of copper reaching Scandinavia in the early second millennium is still unclear and our knowledge about the driving force leading to the establishment of the Bronze Age in southern Scandinavia is fragmentary and incomplete. This study, drawing on a large data set of 210 samples representing almost 50% of all existing metal objects known from this period in Denmark, uses trace element (EDXRF) and isotope analyses (MC-ICP-MS) of copper-based artifacts in combination with substantial typological knowledge to profoundly illuminate the contact directions, networks and routes of the earliest metal supplies. It also presents the first investigation of local recycling or mixing of metals originating from different ore regions. Both continuity and change emerge clearly in the metal-trading networks of the Late Neolithic to the first Bronze Age period. Artifacts in LN II consist mainly of high-impurity copper (so-called fahlore type copper), with the clear exception of British imports. Targeted reuse of foreign artifacts in local production is demonstrated by the presence of British metal in local-style axes. The much smaller range of lead isotope ratios among locally crafted compared to imported artifacts is also likely due to mixing. In the latter half of Nordic LN II (1800–1700 BC), the first signs emerge of a new and distinct type of copper with low impurity levels, which gains enormously in importance later in NBA IA.

## Introduction

This article throws new light on the early phases of metallurgy in southern Scandinavia. It does so by tracking the incoming flows of copper to the region, which ultimately led to the breakthrough of the Nordic Bronze Age c. 1600 BC–a golden epoch boasting such highly sophisticated bronzework as the Trundholm sun chariot. Scandinavia for a long time was marginal to the metallurgical evolution that had begun in the ninth millennium BC in the ancient Near East with the use of beads, pendants and sheet ornaments made from annealed native copper [1, 2]. In central and Mediterranean Europe, the first indications of copper-based technologies date to the later fifth millennium [3–5], initiated through the import of metal ornaments and tools mainly from the Balkan region [6]. According to currently available evidence, however, a regional metallurgy in central Europe does not appear until the beginning of the fourth millennium, with depositions of copper objects such as the Stollhoff hoard [4, 7], the early phases of the Mondsee cultural group [8], and even traces of copper smelting at Brixlegg in Austria around 4000 BC [9].

As early as c. 4400 BC, there are signs of a faint awareness of copper technologies in Scandinavia in the form of rare imports of copper axes into the region’s Late Mesolithic communities [4, 10]. A thousand years later, local metallurgy was likely practiced in the Middle Neolithic Funnelbeaker culture [10–12], only to disappear again subsequently. During most of the third millennium, metallurgy seems absent from the region, even if experiments with casting copper axes and hammering sheet ornaments reappear in Bell Beaker environments in Jutland, 2400–2100 BC. Typology-based studies suggest that the incipient production and import of copper objects in the Funnelbeaker culture around 3500 BC was due to Scandinavia’s involvement in prestige good trading that extended as far as the copper-producing hubs of central and southeastern Europe [8, 13, 14]. A similar explanation may fit the reappearance of copper axes and ornaments c. 2400 BC in the Jutlandish Bell Beaker culture during the first phase of the Late Neolithic (LN I) [12, 15, 16]. At 2000 BC, however, a copper-based technology begins to achieve full economic and social integration in Scandinavia simultaneously with the spread of bronze, or copper with similar properties, across Europe and large tracts of Afro-Eurasia [15, 17–19]. The co-occurrence of these two phenomena is highly significant. Prior to this threshold, metal objects and knowledge of their production had appeared and disappeared several times over the millennia, indicated by, for instance, metallurgical experiments documented at early Funnelbeaker settlement sites [20].

The lack of metal resources can be identified as one major reason for the relatively late *enduring* involvement with metallurgy in Scandinavia. Although copper ores were discovered and exploited in central and northern Scandinavia from the Middle Ages onwards, these indigenous sources were most likely unknown to Bronze Age people and were not exploited [21]. Nordic Bronze Age (NBA) societies were, therefore, completely dependent on exogenous sources from the beginning [15, 21–25].

**Fig 1:**
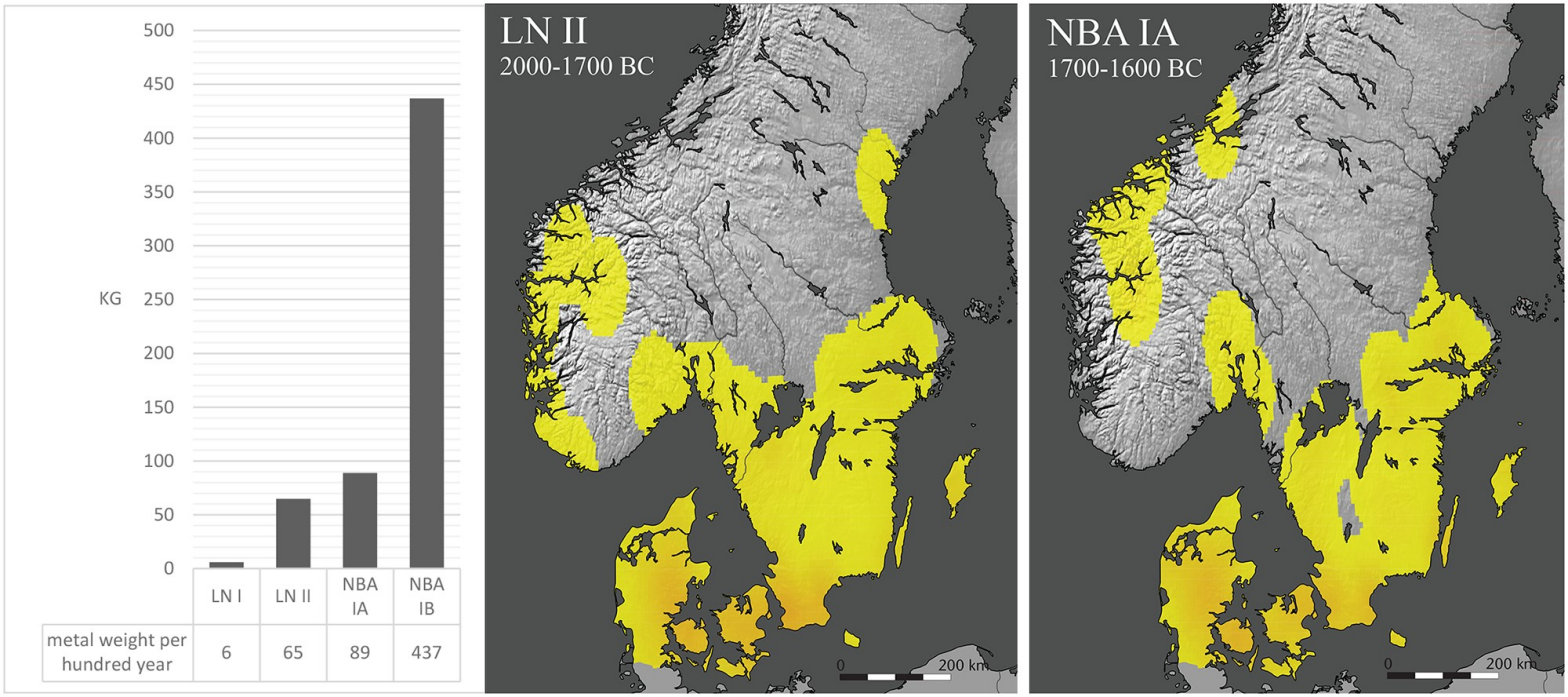
Increase in metal consumption c. 2400–1500 BC. The histogram is based on standard values of metal weight calculated for each main artefact type (i.e. flanged axe, chisel, shafthole axe, and so on [15, 19, 26–44] per 100 year hence compensating for the differing length of the periods. LN II (2000–1700 BC) emerges as the first period of growth. NBA IA (1700–1600 BC) has slightly more metal in circulation. NBA IB (1600–1500 BC) is the breakthrough period, with plentiful metal. The two interpolation maps illustrate the development from LN II (B) to NBA IA (C) on a geographical scale, with orange areas denoting the highest densities. The total number of objects, on which Fig 1 is based, is 1879 from Denmark, Sweden and Norway. Reprinted from [19] based on map images provide by Natural Earth (public domain) under a CC BY 4.0 license, with permission from ArkIT and Helle Vandkilde, original copyright [2017].

The second phase of the Late Neolithic (LN II, c. 2000–1700 BC) can be pinpointed as the crucial turning point, for the very reason that the availability and use of metal increased markedly and grew substantially in the following centuries. With the Scanian Pile hoard as an early highlight showcasing local metalworking activities as early as c. 2000 BC, conclusive evidence now exists for indigenous production of metal objects in southern Scandinavia, more precisely in eastern Jutland, Funen, Zealand and Scania. The high degree of fragmentation in the Pile hoard shows that objects, including rings, were hacked into pieces that would fit into a small crucible and were recast as axes. The next four hundred years laid the foundation for the final breakthrough and subsequent rise of the NBA c. 1600–500 BC with advances in the repertoire and refinement of artifacts, while the consumption of bronze spread northward to central Scandinavia [15, 19] (Fig 1).

**Fig 2:**
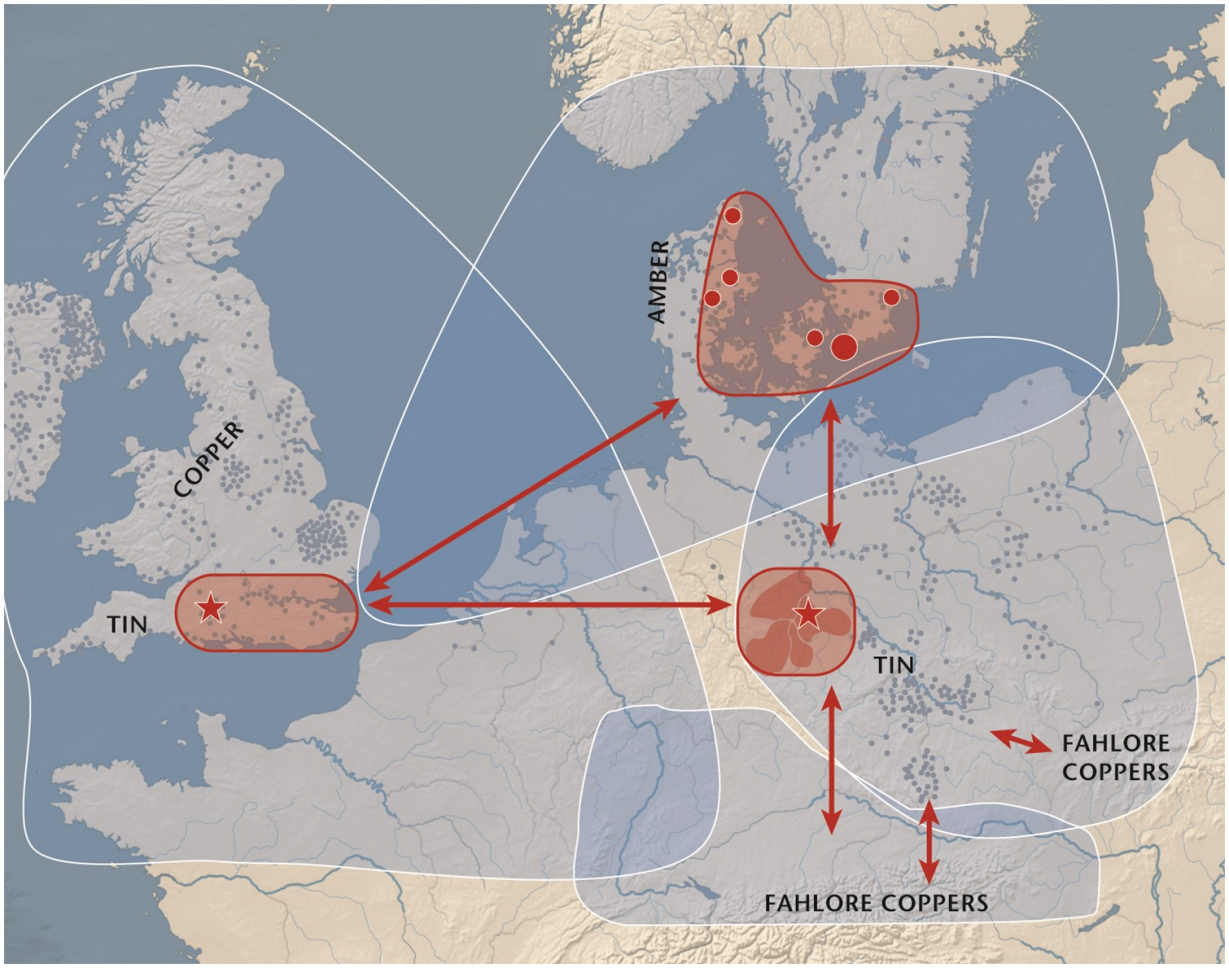
Model of multi-scalar connectivity in northern Europe around 2000 BC from local to super-regional levels and consisting of four overlapping spheres of interaction. The Baltic coast of Mecklenburg-Vorpommern was the pick-up zone for metals to Scandinavia, but the three nodal hub regions–southern Britain with Wessex, eastern Denmark/Scania and the Circum-Harz region with Halle–were directly linked. Remarkably, the main resources on which these hubs relied–namely copper, tin and amber–were located outside their geographical precinct. Map images are provided by Natural Earth (public domain) under a CC BY 4.0 license. The map, first published by H. V. [19], is with permission reworked by L. H. and H. V. using the software Adobe Illustrator. Contains data from [15, 49–59].

The metal supply to southern Scandinavia held the key to creative cultural achievement in the far northwest of the Bronze Age world. Vandkilde’s recent publication [19] has provided a good overview of scalar connectivity 2000–1700 BCE (Fig 2). Interpretations of recent results in trace element and isotope science have identified a diverse and geographically shifting network of long-distance trading routes over the entire duration of the Bronze Age up to c. 500 BC [21, 25, 45, 46] although many of the claimed relationships between ores and artefacts were contested [47, 48] and were shown to be valid only in much later phases of the Bronze Age [45]. This interpretation was necessarily painted with a broad brush as it covered 1,500 years, and the crucial early part of this period was underrepresented in the data used in these publications (only seven data sets from LN II–NBA IA apart from the project presented here [46]), leaving the precise provenance of copper reaching Scandinavia in the early second millennium still as an open question. By comparison, the present study draws on a much larger number of both trace element and isotope analyses of copper-based artifacts, amounting to 210 samples and representing 50% of all existing metal objects known from this period in Denmark.

Because the objects investigated in this project date to 2000–1600 BC comprising Late Neolithic (LN II) and earliest Bronze Age (NBA IA), it has extraordinary potential to answer questions regarding the final breakthrough of the NBA c. 1600 BC–not only about the specific sources of the imported copper, but also the ways this metal was used locally. The result therefore offers an improved understanding of long-term developments and also of the degree to which shifts in metal supplies coincided with culture-historical watersheds. The article thus substantially improves our understanding of the hitherto vaguely understood metal trading networks and metallurgical developments that led to the NBA. It also investigates, for the first time, the question of local recycling, or mixing, of metals originating from different ore regions [47].

## Methods

Sampling and analyses were performed with a view to limit as much as possible destructive interventions on Bronze Age artifacts. Archived samples collected within the Stuttgarter Metallanalysen project (SAM) in the 1970s were therefore used [17, 60, 61]. Sample preparation included tracing and sorting the samples taken from C. Cullberg for the SAM project [62] between 1959 and 1962 of Danish artifacts and aligning specimens and artifacts (S1 Table). For minor and trace element analysis corrosion material mixed with the drill shavings was carefully removed under a microscope, if necessary. The analysis of minor and trace elements was accomplished with an energy-dispersive X-ray fluorescence spectrometer (EDXRF). Concentrations of the elements Mn, Fe, Co, Ni, Zn, As, Se, Ag, Cd, Sn, Sb, Te, Au, Pb, and Bi were measured at the CEZA in Mannheim, Germany, with an ARL Quant X (Thermo Scientific) instrument. The samples were placed on a 20-position sample changer, which is especially useful for drilled samples. The samples were measured in two exposures of 600 seconds each following a modified version of the procedure by Lutz and Pernicka [63]. Eighteen samples could thus be measured in one run within less than eight hours. Two reference materials obtained from the Bundesanstalt für Materialprüfung in Berlin (BAM211 and BAM376) were included in each run. As indicated in the database (S2 Table) the detection limits are 0.05% for Fe, around 0.01% for Co, Ni, and As, and around 0.005 for Ag, Sb, Sn, Au, Pb, and Bi. Mn, Cd, Se, and Te were also measured but were below 0.005% in all samples. Zn was below the detection limit of 0.1% in all samples.

The calculations for the amount of metal in circulation in the Late Neolithic and NBA IA are based on studies of a large number of inventories performed in Denmark, northern Germany [15, 26–40, 64], and Sweden [15, 41]. In all, 210 analyses were accomplished within the present project, of which 155 are from artifacts dating to LN II. The remaining data illustrating general tendencies are taken from the SAM and SMAP projects [17, 61, 65] and small-scale local studies [19, 66] with different devices. The precision of the SAM data is with ± 30% relatively low but they are quite accurate [67]. However, some trace elements need to be considered with caution: high As values are significantly lower in the SAM dataset. Sb and Ag are only comparable at concentrations above 0.35%, and Ni has a good comparability at concentrations between 0.12 and 1.2%. Outside this range larger deviations occur.

The determination of the lead isotope ratios was performed with a multiple-collector inductively-coupled plasma mass spectrometer (MC–ICP–MS) at the Curt–Engelhorn Center for Archaeometry (CEZA) in Mannheim, Germany. The instrument used was a Thermo Scientific Neptune Plus mass spectrometer. For the measurements solutions with a lead concentration of 100 ng/ml were prepared after dissolution of the solid samples and chemical separation of the lead. For the separation of lead the samples were rinsed with dilute HNO_3_ to remove surface contamination and then dissolved in half-concentrated HNO_3_ in an ultrasonic bath (70°C) for several hours. Insoluble residues were removed by decantation from the resulting solution, which was then diluted with deionised water [68]. Columns were prepared with PRE filter resin and Sr resin, and preconditioned with 500μl 3N HNO_3_ before the solution was added. The matrix was eluted in four steps, using HNO_3_, and then the Pb was eluted using HCl. After drying (48h), thallium was added to the sample solutions to monitor and correct the internal mass fractionation [69]. Standard solutions were measured after every fourth sample and intensive cleaning of the tubing was conducted after every sample. The isotope ratios measured were ^208^Pb/^206^Pb, ^207^Pb/^206^Pb, and ^206^Pb/^204^Pb, with relative uncertainties of less than 0.01 for the first two ratios and 0.03% for the last. The ratios ^208^Pb/^204^Pb and ^207^Pb/^204^Pb were calculated from the other ratios.

## Material

**Fig 3:**
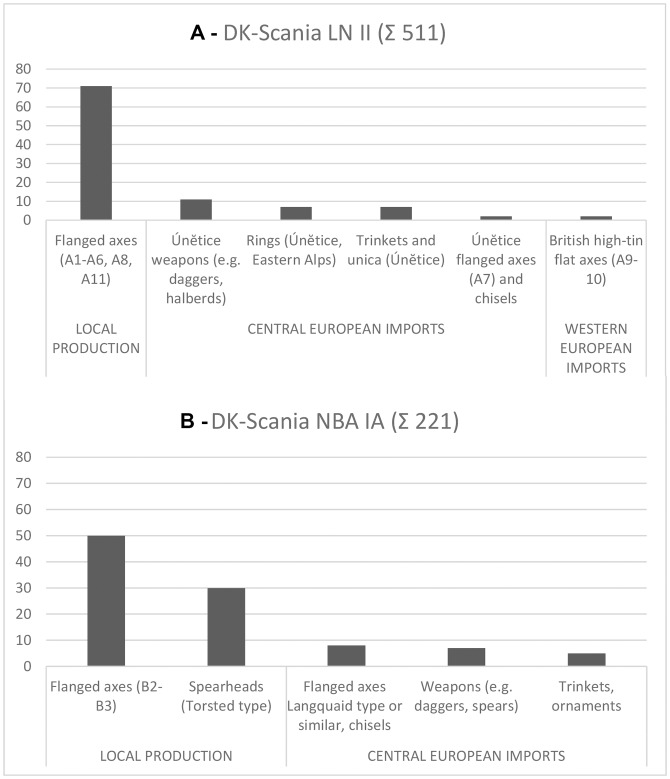
Metalwork in LN II (Fig 3A) and NBA IA (Fig 3B) quantitatively arranged according to the main types, local production or foreign import. Although local production is by far the most common, it includes a category of hybrids. This indicates that imports were routinely remelted and some were recast in an ‘in-between’ style. This hints at copper mixing. Regional inventory studies were used to calculate the total number of objects in circulation [15, 26–41, 64].

Right from the beginning, the number of locally produced objects exceeded those of imported objects in southern Scandinavia. Axes–functioning as weapons as well as tools–constitute by far the most common category of metal artifact produced in Scandinavia during the four hundred years of the earliest Bronze Age (Fig 3), and these therefore provide the critical archaeological data for archaeometallurgical analysis. The acquired samples are partly legacies of the *Stuttgarter Metallanalysenprojekt* [17, 60–62, 65], while in a number of cases new samples were obtained from objects in the National Museum of Denmark, Copenhagen, in order to obtain a balance between different object types: in particular, between imported axes and the predominant local group of axes. The majority of samples (141 counts) derive from LN II (2000–1700 BC) and a further 50 samples from NBA IA (1700–1600 BC). Although fifty samples may appear as a small fraction, they nevertheless represents 28% of the known artifacts; 62% of known axes from the relatively brief NBA IA period were also available for analysis, in addition to several spearheads. All samples stem from distinct morphology-defined types, which are easy to divide into locally made and imported [15]: this division is a key component of the archaeometallurgical study described below. Some local axe types clearly express their localness through style, while others retain foreign traits due to having direct foreign antecedents or inspirations (e.g. pseudo-British axes are local versions of high-tin British axe imports, and the large Virring type axes are distinctly local versions of central European Langquaid type axes; see S1 Fig). Such artifact groups often have a confined geographical distribution, as do technical and ornamental traditions [70].

In consequence, artifacts of a specific characteristic style or technological feature can be provenanced to particular regions and cultural groups. Larger-scale geographical patterns thus help to distinguish local products from foreign imports. For instance, axes of British type and Langquaid type found in Denmark are classified as imported because of their distinct morphology and their main distribution area in the British Isles and central Europe respectively [51, 71]. In contrast, local axes such as the Gallemose and Torsted-Virring types are characteristically Nordic in their style and geographical distribution. The scientific provenancing of copper through geochemistry and isotopy is thus profoundly enriched by the substantial typological knowledge that is a cornerstone of this study.

## Results

### The evidence of typochronology

In **LN II (2000–1700 BC)** as many as 71% of the artifacts can be classified as locally produced axe forms (Fig 3A). The remaining 29% can be divided between a small group of British-developed bronze flat axes (2%) and a large group of imports from the central European Únĕtice culture (27%), notably comprising halberds, metal-hilted daggers, and rings in addition to rare items of gold, silver or bronze. British axes had arrived in Scandinavia already by the turn from the third to the second millennium BC, with isolated specimens of Killaha–Migdale type axes occurring on the west coast of Denmark already in the final LN I [19]. New arrivals during LN II include large and spectacularly decorated axe-heads belonging to the Ballyvalley–Scrabo Hill type of developed British bronze flat axes and boasting a very high tin content [49, 51, 72]. Early Scandinavian production of axes is strikingly diverse and features several varieties, testifying to slow formation of style and ongoing experimentation. The local tradition of ‘Pile type’ axes is nevertheless prominent, with its distinctly parallel-sided curved shape and, in many cases, the characteristic decoration of multiple lines on the blade (A3, A4 –Gallemose type and Vaerslev type axes [15]).

A minority group of locally made axes are typological ‘in-betweens’–that is, not entirely local and not entirely foreign (see S1 Fig). Pseudo-British axes (A8 [15]) emulate the British high-tin developed flat axes (A10 [15]). The Emmen and Hjadstrup low-flanged axes (A1-A2 [15]) recall a general west European trapezoidal axe tradition, perhaps including the Swiss Neyruz type [73, 74]. The Store Heddinge and Æbelnæs types (A5-A6) of low-flanged axes resemble the Únĕtician Saxo-Bohemian axe with its distinct waist [15, 54, 75]. The morphology of the hybrids, however, shows proportions deviating from the foreign role models, and quite often the Nordic multi-line decoration has been applied. This phenomenon of hybridization suggests that an attractive foreign axe was at some point recast in a shape that retained its original traits but also added local-style traits of form or decoration. Importantly, this in turn suggests that copper with potentially different provenance may have been mixed to a certain extent, as it would often be necessary to add new metal to the original charge when casting an item. Local metalwork production integrated inspiration from western Europe (including the British Isles) and central Europe, most particularly the Únĕtice society, which extended from the Baltic Sea to the foothills of the Alps with a west–east extension from the Aller river (central Germany) to the Warta river (Great Poland). Crucially, this is a strong indication of where to locate prevailing routes of metal trading to Scandinavia, if not the exact mines favored (Fig 2).

In the prologue to the Nordic Bronze Age proper, **NBA IA (1700–1600 BC)**, the proportion and variety of foreign imports decrease markedly. The local Nordic production now amounts to at least 80%, and all or most other metal objects derive from central Europe, most likely the Únӗtice culture in its final phase. Probable imports comprise large spearheads, chisels, daggers, and various trinkets in addition to flanged axes with elongated body and spoon-shaped, spatula-shaped or half-circular cutting-edge, such as the Langquaid type. By comparison, it is difficult to pinpoint objects with a clear west European or British appearance (Fig 3B). Locally made products are by now remarkably uniform in their style and hence easy to recognize as a Nordic tradition. This uniformity may suggest the involvement of a limited number of professional workshops.

Nevertheless, the Nordic production of flanged axes, and now also spearheads, adheres to stylistic trends shared with the rest of temperate Europe in the seventeenth century BC: various types of flanged axes with pronounced cutting-edge, and the first socketed spearheads to equip the warrior. The Nordic Torsted-Virring axes (B2-B3) notably emulate the central European Langquaid type [74] flanged axe (B4). Metal trading routes to Scandinavia still involve central Europe, but artifact typology does not clearly reveal which part. Given the general international character of metalwork in temperate Europe throughout this period, it is possible that the British Isles were still involved in the trading of metals to Scandinavia during NBA IA.

### The evidence of metal analysis: Trace element compositions (TE)

**Fig 4:**
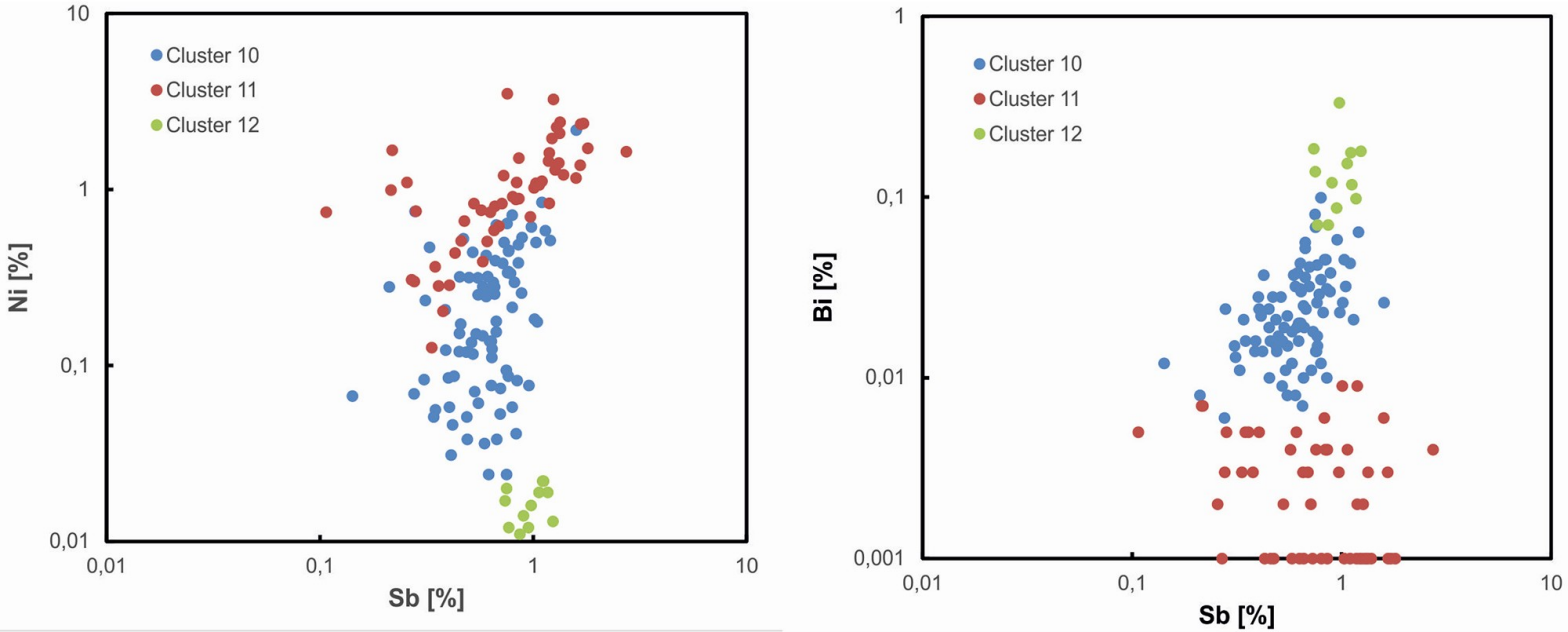
Bivariate analysis of Ni/Sb and Bi/Sb values. The three fahlore types of clusters 10–11 stand distinct. The Ni-free copper (cluster 12) is a separate type, while the medium-Ni (cluster 10) and high-Ni (cluster 11) copper show overlap, indicating a common ore source or possible mixing.

Using the typology-based results as a scaffold, further investigations were made using metal analysis. The following average-link cluster analysis (cf. Table 1) based on logarithmic concentrations of arsenic (As), antimony (Sb), silver (Ag), nickel (Ni) and bismuth (Bi), executed on an extended dataset of 450 objects dating from LN II to NBA IB, disclosed thirteen compositional groups that reveal chronological continuity as well as breaks. High-impurity copper–so-called fahlore copper–is with 88% dominant in LN II artifacts. The high impurity levels of the fahlore copper allow us to differentiate three different copper types (Fig 4).

**Table 1:** Average trace element composition in mass percent of the major clusters defined via average-link cluster analysis based on logarithmic concentrations of arsenic (As), antimony (Sb), silver (Ag), nickel (Ni) and bismuth (Bi), executed on an extended dataset of 450 objects dating from LNII to NBA IB.

		Co	Ni	As	Ag	Sn	Sb	Pb	Bi
Cluster 4	Average	0.017	0.46	0.51	0.040	7.6	0.108	0.14	0.001
(39 samples)	Median	0.015	0.44	0.47	0.025	7.6	0.080	0.048	0.001
	Quantil 0.1	0.010	0.24	0.24	0.011	6.0	0.031	0.008	0.000
	Quantil 0.9	0.029	0.72	0.78	0.100	9.4	0.199	0.30	0.003
Cluster 5–7	Average	0.002	0.024	0.99	0.029	0.91	0.026	0.34	0.010
(7 samples)	Median	0.000	0.022	1.12	0.026	0.005	0.017	0.025	0.004
	Quantil 0.1	0.000	0.017	0.42	0.012	0.002	0.010	0.013	0.001
	Quantil 0.9	0.005	0.033	1.40	0.052	1.64	0.060	0.66	0.025
Cluster 8	Average	0.009	0.107	0.150	0.008	8.4	0.021	0.013	0.001
(21 samples)	Median	0.010	0.072	0.125	0.008	8.4	0.012	0.007	0.001
	Quantil 0.1	0.000	0.020	0.056	0.004	5.7	0.005	0.002	0.000
	Quantil 0.9	0.013	0.210	0.252	0.012	12.1	0.052	0.035	0.001
Cluster 10	Average	0.009	0.27	0.64	0.79	2.36	0.64	0.023	0.026
(99 samples)	Median	0.010	0.181	0.60	0.83	1.33	0.64	0.008	0.022
	Quantil 0.1	0.000	0.051	0.36	0.40	0.05	0.37	0.003	0.011
	Quantil 0.9	0.010	0.53	0.95	1.09	6.90	0.92	0.026	0.045
Cluster 11	Average	0.028	1.13	0.58	0.74	1.94	0.88	0.016	0.003
(53 Proben)	Median	0.020	0.99	0.40	0.80	0.50	0.80	0.006	0.002
	Quantil 0.1	0.010	0.32	0.21	0.28	0.10	0.28	0.002	0.001
	Quantil 0.9	0.070	2.21	0.94	1.06	5.10	1.55	0.022	0.006
Cluster 12	Average	0.009	0.016	1.52	1.39	0.058	0.970	0.018	0.144
(12 samples)	Median	0.010	0.017	1.55	1.28	0.008	0.962	0.011	0.129
	Quantil 0.1	0.010	0.012	1.15	1.08	0.002	0.751	0.002	0.072
	Quantil 0.9	0.010	0.022	1.89	1.59	0.093	1.165	0.029	0.184

Two fahlore clusters predominate in the **LN II 2000–1700 BC** assemblage; they share minor components, but differ in their nickel concentrations. High-Ni fahlore copper (up to 1% Ni, cluster 11) is characterized by Sb > Ag > As at concentrations of more than 0.5%. The medium-Ni fahlore copper (cluster 10) accounts for nearly two thirds of the artifacts and contains Ni values of around 0.2% and high values of Ag > Sb = As. Within both coppers groups artefacts with tin values of up to 2% appear. The third and smallest group of the fahlore copper (cluster 12) is characterized by the absence of nickel (up to 0.03% but less), and displays a trace element pattern of As = > Ag > Sb at high concentrations with a median of arsenic at 1.55%, more than 0.1% bismuth, and Sn only as minor TE. The trace element investigation additionally revealed several minor groups, some of which had already been identified by the SAM project [17, 61].

A small group of axes dating from 2000–1700 BC can be shown to consist of non-fahlore low-impurity copper with only low concentrations of nickel (0.1%); around 6% of the artifacts analyzed in LN II consist of this low-impurity copper, where the different levels of arsenic and silver (and tin, although it was not used for classification) explain the distribution into five clusters, 1–3 (3%), 8–9 (3%). It is conceivable that these five clusters actually belong to one type of copper with larger internal variation than the clusters discussed before. Unlike most LN II metal, these axes contain significant proportions of tin, commonly between 8 and 13%, with low impurities of Ni and As (cluster 8 and 9). This high tin content is indicative of deliberately alloyed bronze objects. Copper alloys with high tin contents are relatively rare in temperate Europe 2000–1700 BC, with the British Isles as the only place boasting predominantly high-tin bronzes during this period [76–78].

Importantly, it is already during the latter half of Nordic LN II, 1800–1700 BC, that the first sign of a new and distinct minor-impurity copper emerges, most likely from copper ores that predominantly consist of chalcopyrite, as expressed in cluster 4. The median values of this copper group follow an impurity pattern of Ni = As > Sb and, occasionally, traces of silver. A closer examination of the large cluster 4 reveals several subgroups as already indicated by the difference between the interdeciles in Table 1. One specific subgroup, defined by an impurity pattern of Ni > As, Ag, Sb and Sn between 6–11%, represents 6% of the LN II artifacts examined.

**Fig 5:**
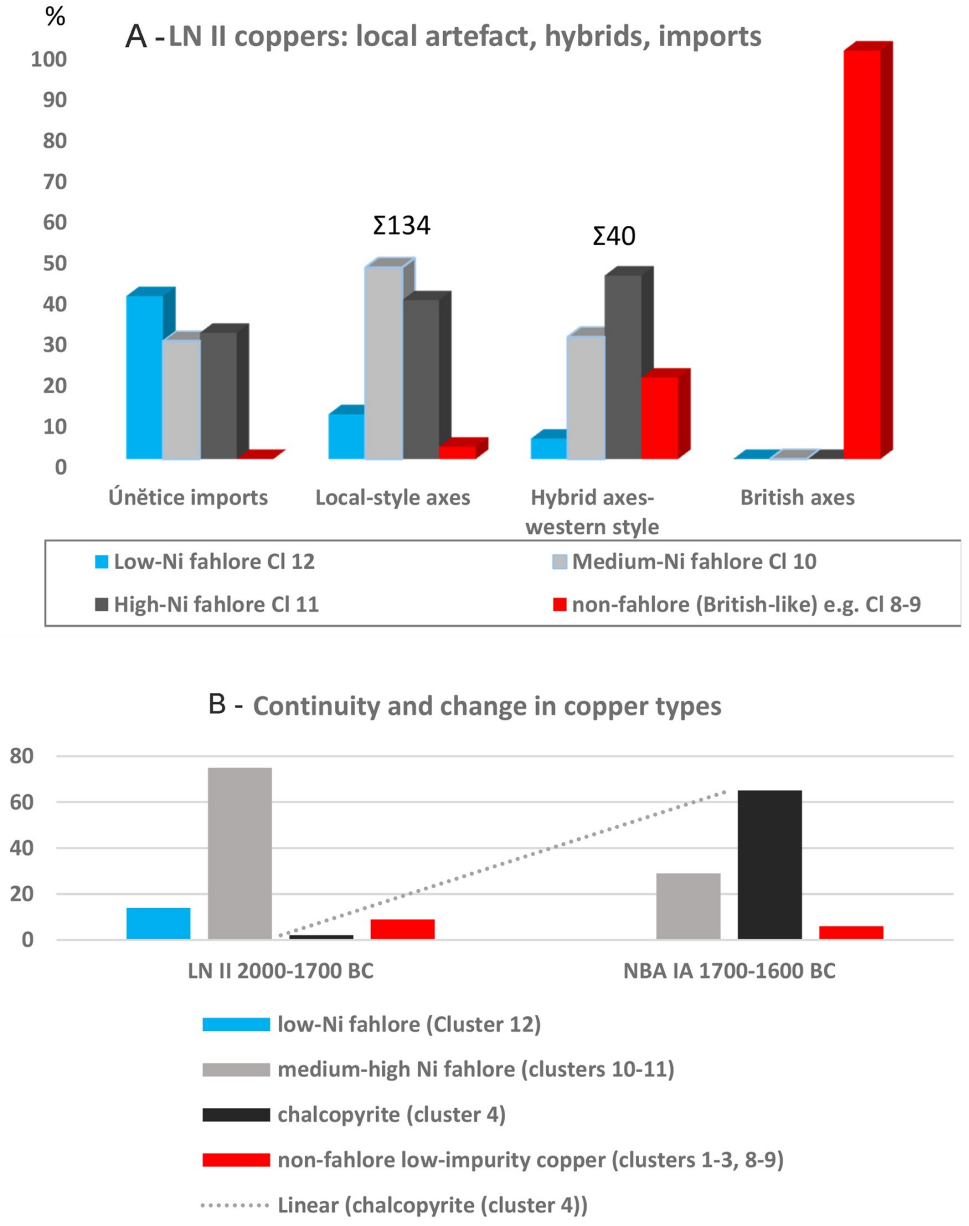
Copper types and their association with artifact styles. (A) The primary copper groups in Scandinavian LN II and their association with imported, local-style and hybrid artifacts. Únĕtician and British inputs differ markedly from one another, as is evident in both artifact style and copper type. (B) Continuity and change in copper type 2000–1600 BC in Scandinavia. Ni-fahlores and low-impurity copper occur throughout the period. Ni-free fahlore is a LN II phenomenon, while the minor-impurity copper of cluster 4 dominates in NBA IA.

When drawing on a larger Scandinavian sample of 280 analyses for LN II and taking into account the artifacts’ status as local, import or hybrid, the trace element analysis revealed an informative pattern (Fig 5A). Firstly, Únĕtician imports show an almost equal distribution of all three fahlore types, although the Ni-free variety is the most prominent. Significantly, the majority of rings analyzed (Únĕtician and a few east Alpine forms) (cluster 12) belong to this distinctive copper, which is equivalent to so-called Ösenring copper, often thought to be of east Alpine provenance [9, 65]. This Ni-free Ösenring copper is the prevailing metal in the Scanian Pile hoard dating to c. 2000 BC, and it is the earliest in a series of spectacular hoards [19]. Secondly, local Nordic-style axes, though sometimes associated with Ösenring copper, are primarily made from the two Ni-fahlores. High-Ni fahlore copper (cluster 11) tends to be slightly more common than the medium-Ni variety (cluster 10), while the Únĕtician imports show the opposite tendency. Thirdly, British type non-fahlore copper–typically low-impurity and with high tin (especially clusters 8–9)–is traceable among the local-style axes, becomes significantly more prominent among hybrid western-style axes, and is completely dominant in the small but spectacular group of British axes. The equal prominence of medium and high-Ni fahlore, also characterizing the local-style axe group, undoubtedly indicate that the hybrid axes of western style were made in Scandinavia. These observations have repercussions for the questions of the degree of local copper mixing and the location of primary regions of provenance for these copper groups.

The copper of **NBA IA, 1700–1600 BC**, is less diverse with regard to trace element composition. The majority of the artifacts (68%) now consist of the minor-impurity copper of cluster 4, with a consistent impurity pattern of Ni = As > Sb (cluster 4). Most local artifacts are now made from this new type of copper (cf. Fig 5B). However, a large proportion of the artifacts (28%) are still made of the familiar Ni-fahlore copper (clusters 10 and 11). A very similar bipartite situation was found to prevail in the recently discovered contemporaneous hoard of almost 800 rib ingots at Oberding in Bavaria [79] where the minor-impurity copper was related to the Mitterberg region in Austria. By NBA IA the Ni-free fahlore, Ösenring copper, so prevalent at the very outset of LN II, has disappeared. In addition to this, the deviant non-fahlore low-impurity clusters with high tin content are still recognizable in the data and may be related to the same British sources as previously. The objects produced now from this low-impurity copper are of Nordic style and technique, and therefore produced in Scandinavia. Use of the above data to track and visualize the development in copper groups shows clear continuity as well as change (Fig 5B), which is in accordance with the evidence of typochronology as well as lead isotopy.

### The evidence of metal analysis: Lead isotopic signatures and ranges

**Fig 6:**
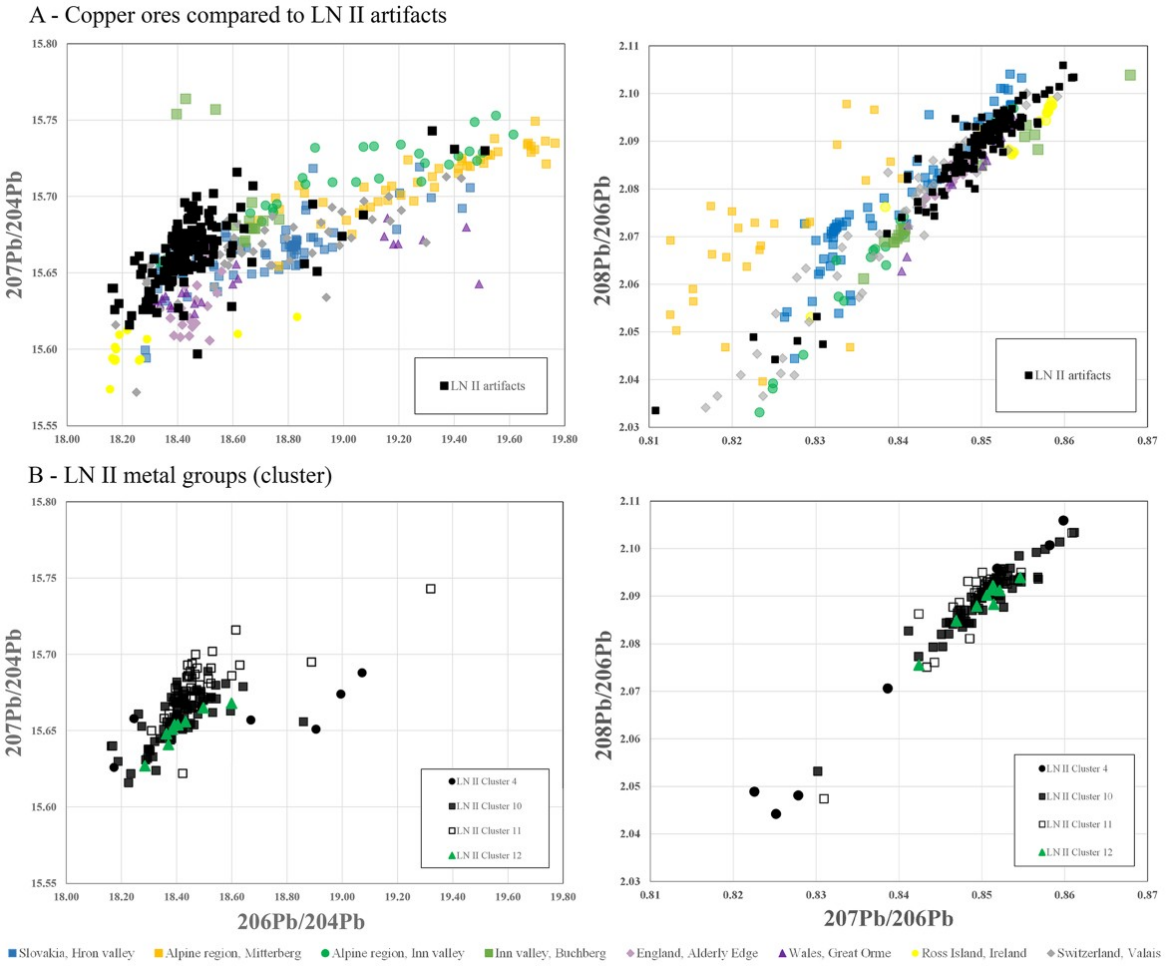
Lead isotope ratios of the artifacts from LN II compared with possible ore sources. A) compares the most likely copper sources with the complete dataset for LN II while B) displays the different clusters during LN II. The ore data are from: Mitterberg ore district [48]; Hron valley, Slovakian Ore Mountains [59, 80]; Inn Valley, Alpine region [9]; Buchberg, Inn Valley, Alpine region [81]; Great Orme mining region, Wales [82–86]; Alderley Edge mining region [85, 86]; Valais valley, Switzerland [87]. The analytical uncertainties are comparable with the size of the symbols.

**Fig 7:**
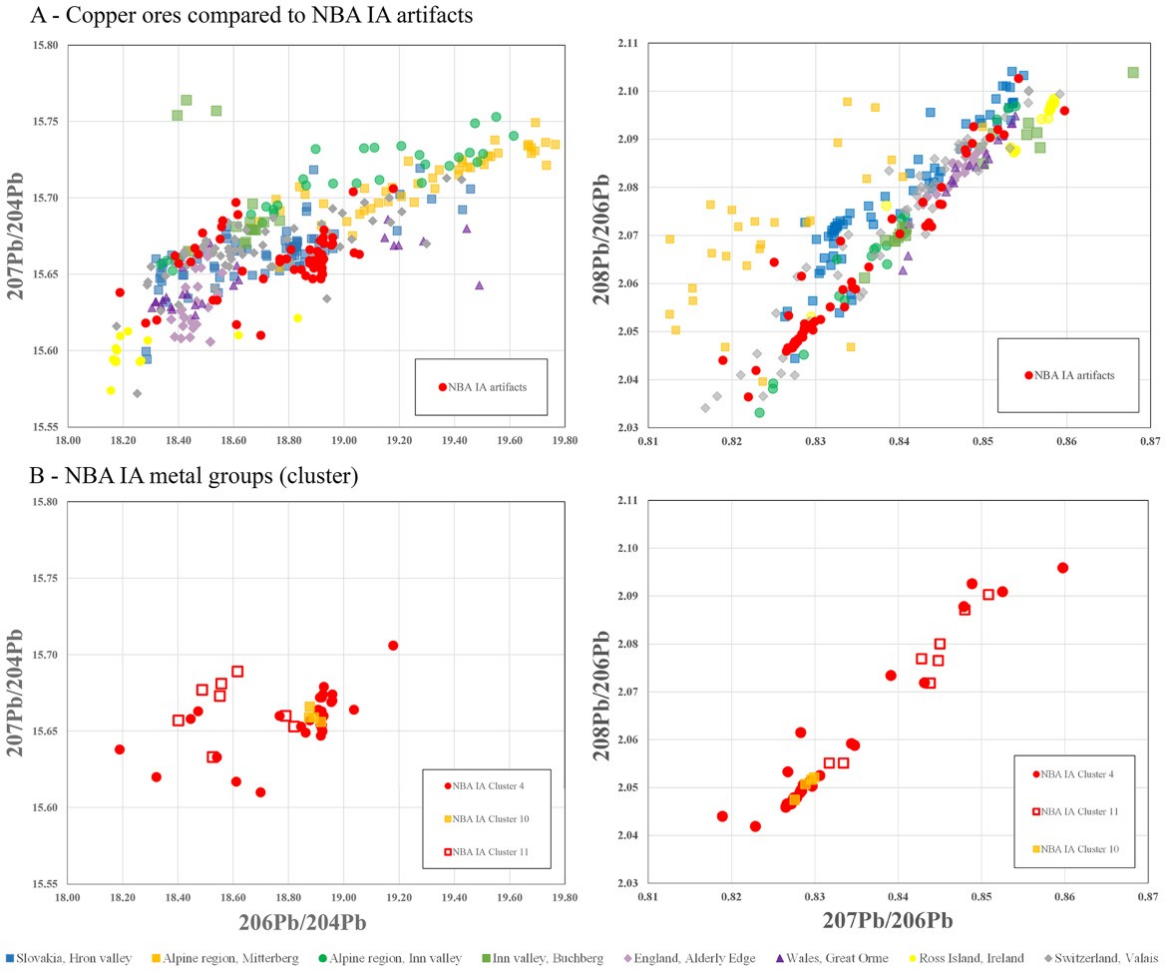
Lead isotope ratios of the artifacts from NBA IA compared with possible ore sources. A) compares the ore data with the complete dataset from NBA IA while B) displays the different copper groups during NBA IA. The ore data are from: Mitterberg ore district [48]; Hron valley, Slovakian Ore Mountains [59, 80]; Inn Valley, Alpine region [9]; Buchberg, Inn Valley, Alpine region [81]; Great Orme mining region, Wales [82–86]; Alderley Edge mining region [85, 86]; Valais valley, Switzerland [87]. The analytical uncertainties are comparable with the size of the symbols.

**Fig 8:**
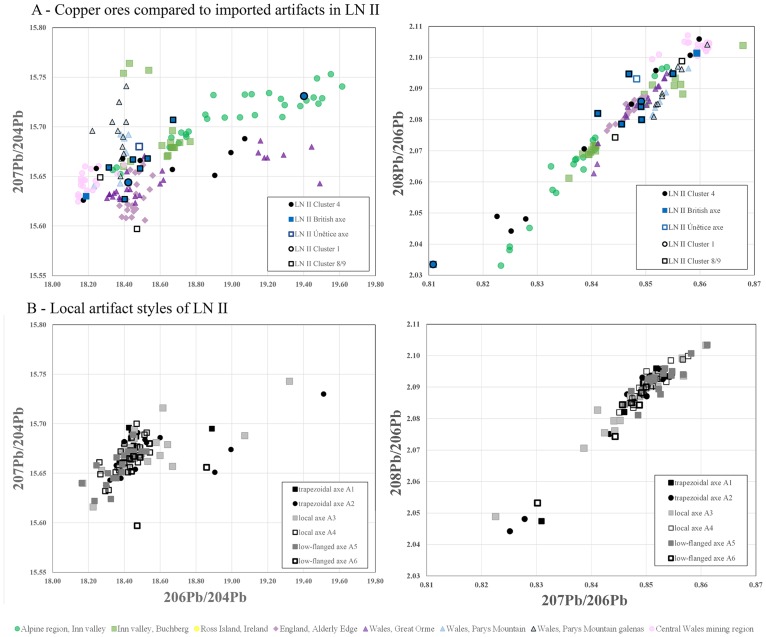
Lead isotope ratios of artifacts of secured foreign origin during LN II. The typologically British and Únĕtician artifacts (A) show a wider distribution than the typologically local-crafted artifacts (B). The comparison with ore bodies having pure copper highlights the comparability of British ores with British artifacts, more precisely with Great Orme metal. The ore-related data are from: Ross Island [85, 86, 88]; Great Orme mining region, Wales [82–86]; north and central Wales mining regions [86, 89]; Alderley Edge mining region [85, 86]; eastern Alpine ores [9, 81].

**Fig 9:**
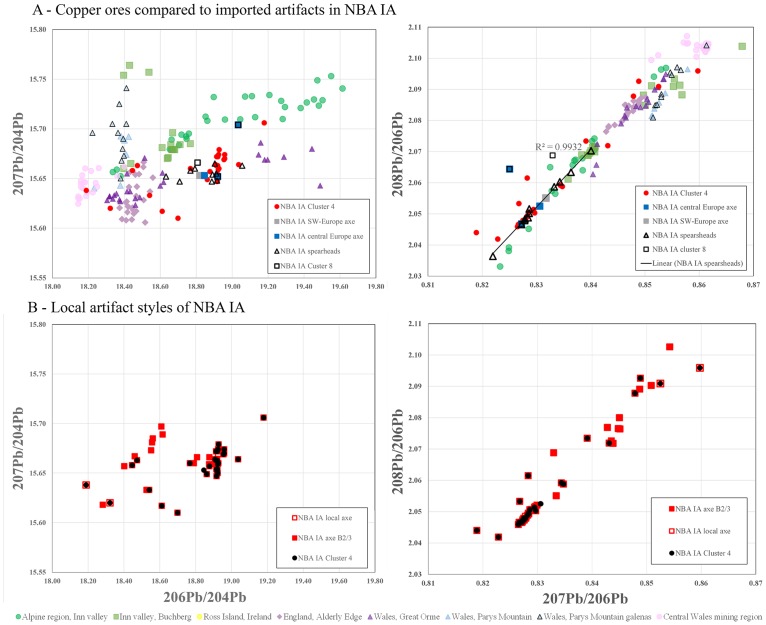
Lead isotope ratios of artifacts of secured foreign origin during NBA IA. The few typologically foreign artifacts (A) can be still correlated with cluster 8 metal, though different in type from the British ores. The alignment of new artifact types including spearheads supports the assumption of mixing. The typologically local-crafted artifacts (B) are consistent with cluster 4 metal and in this case can be correlated with British ores. The ore-related data are from: Ross Island [85, 86, 88]; Great Orme mining region, Wales [82–86]; north and central Wales mining regions [86, 89]; Alderley Edge mining region [85, 86]; eastern Alpine ores [9, 81].

In the evaluation of the lead isotope ratios, the results of the cluster analysis were taken into consideration in order to detect chronological and/or regional variations, as presented above. In a first step, the isotope ratios of the artifacts were plotted in accordance with their chronology and their above-defined clusters; in a second step, the artifact types were separated within each cluster. The plots (Figs 6 to 9) follow the convention (above) of illustrating several varieties and combinations of lead isotope ratios against the background of potentially relevant ore bodies (S2 Fig), of which several can be dismissed on various grounds (see Discussion).

Isotope plots show a remarkably clear differentiation between LN II and NBA IA data, corroborating the patterns identified with trace element concentrations shown above. The majority of the artifacts measured stand together as a group with very similar isotope signatures (Figs 6 and 7). In LN II, locally crafted artifacts occur mostly within a range of very similar signatures. By contrast, outliers with isotopically more diverse values are identified mainly as foreign imports, notably including the artifacts in cluster 4 (Fig 8). This distinct centralization of a major part of the dataset (i.e. the locally crafted artifacts, Fig 8B) indicates that mixing of metal from several sources (artifacts or ores) took place in the course of the local metalwork crafting. However, this centralized pattern changes markedly in NBA IA, where the few distinct imports (Fig 9) are isotopically lined up with locally produced artifacts. This could be interpreted as indicating mixing of only two major sources. In this case, imported artifacts, namely British and Austrian metal, may be considered as endpoints in a mixing line of extensively reused older artifacts which were transformed into new artifact types (Fig 9B). This pertains especially to socketed spearheads, a new weapon that appears for the first time in NBA IA (Fig 9B).

A number of conclusions emerge. Firstly, the few artifacts produced from the minor-impurity copper of cluster 4 in LN II are likely to represent a new type and source of incoming copper, one that later in NBA IA gains enormously in importance. In LN II this novel copper type is characterized by very diverse isotopic values, while in NBA IA the range of Pb-isotope ratios is restricted to a degree that could either be explained as a new and less variable source or as homogenization due to mixing (Figs 6 to 9). Secondly, the change from LN II to NBA IA in the isotope ratios of the medium-Ni fahlore copper (cluster 10) is significant, especially in the ^208^Pb/^206^Pb to ^207^Pb/^206^Pb ratios. In LN II, this Ag>Sb = As>Ni fahlore displays ^207^Pb/^206^Pb ratios from 0.84234 to 0.85447 and ^208^Pb/^206^Pb ratios from 2.07720 to 2.09850. In the subsequent NBA IA period, by contrast, the artifacts display much lower values: ^207^Pb/^206^Pb ratios around 0.828 and ^208^Pb/^206^Pb ratios around 2.05. This could indicate a shift during NBA IA to another medium-Ni fahlore copper source, though within the same ore body. Thirdly, a lesser and more diffuse change in isotope values characterizes the Ni-rich fahlore group of cluster 11. The exploitation in NBA IA of a new branch, or deeper level, of the same ore deposit may explain this change. Fourthly, the isotopic similarities between the NBA IA medium-Ni fahlore (cluster 10) and parts of contemporaneous artifacts made of the minor-impurity copper of cluster 4 seem to indicate mixing of artifacts with artifacts or ores. Fifthly, with regard to lead isotope ratios, the Ni-free Ösenring copper in the Pile hoard has been shown to be compatible with copper ore from the eastern Alps [90]. The Inn Valley and Buchberg in particular is the most likely possibility; the Slovakian Ore Mountains are an alternative (Figs 6 and 7), but one that can be ruled out by comparison of the trace element patterns.

The isotope plots confirm the British connection and the local reuse of British metal. In LN II, the morphologically distinct British axes fall in several clusters, mainly clusters 8–9 and the non-fahlore low-impurity copper of clusters 1–3 and 5 (together with a proportion of the halberds). The particularity of the British axes/metal is reflected in the isotope plots of ^208^Pb/^206^Pb and ^207^Pb/^206^Pb ranging between 2.0743–2.0988 and 0.84435–0.85667 and ^206^Pb/^204^Pb and ^207^Pb/^204^Pb ratios ranging between 15.649–15.658 and 18.403–18.499. These values are more diverse than those of the local artifacts (see Fig 8). Remarkably, locally made axes of the Gallemose (A3) and Store Heddinge (A5) types sometimes display quite similar isotope ratios, and in these cases one can therefore assume that British axe metal was directly reused for local products. In NBA IA, however, British-style artifacts have disappeared. The above-mentioned isotope ratios of low-impurity copper that in LN II were clearly linked to British axes have in NBA IA changed character, and they now exhibit more divergent values than the British–Irish ores (Fig 9).

## Discussion

### Scandinavia’s early metallurgy: In pursuit of copper mixing

Pinpointing the provenance of the copper reaching Scandinavia in more detail requires taking a closer look at the central question of mixing–whether and to what degree this is a phenomenon in the start-up phases of the Bronze Age. Above, the hybridization of style indicated targeted mixing of copper of different provenance, as do the deviant trace element patterns in imports compared to local items. The multi-methodological approach implemented here suggests an intended mixing of artefact metal in the production of the earliest copper-based artifacts instead of the proposed use of copper and tin ingots suggested for the Swedish bronzes [25]. Furthermore, some copper clusters (cf. Figs 6 to 9) have a wide range of lead isotope ratios combined with a similar trace elemental composition, while others are confined in both sets of geochemical parameters. In particular the large Ni-fahlore copper groups of clusters 10 and 11 and the minor-impurity copper of cluster 4 exemplify a known problem: the mathematical configuration of clusters, their in-group diversity, and their relation to artifact types [91, 92].

**Fig 10:**
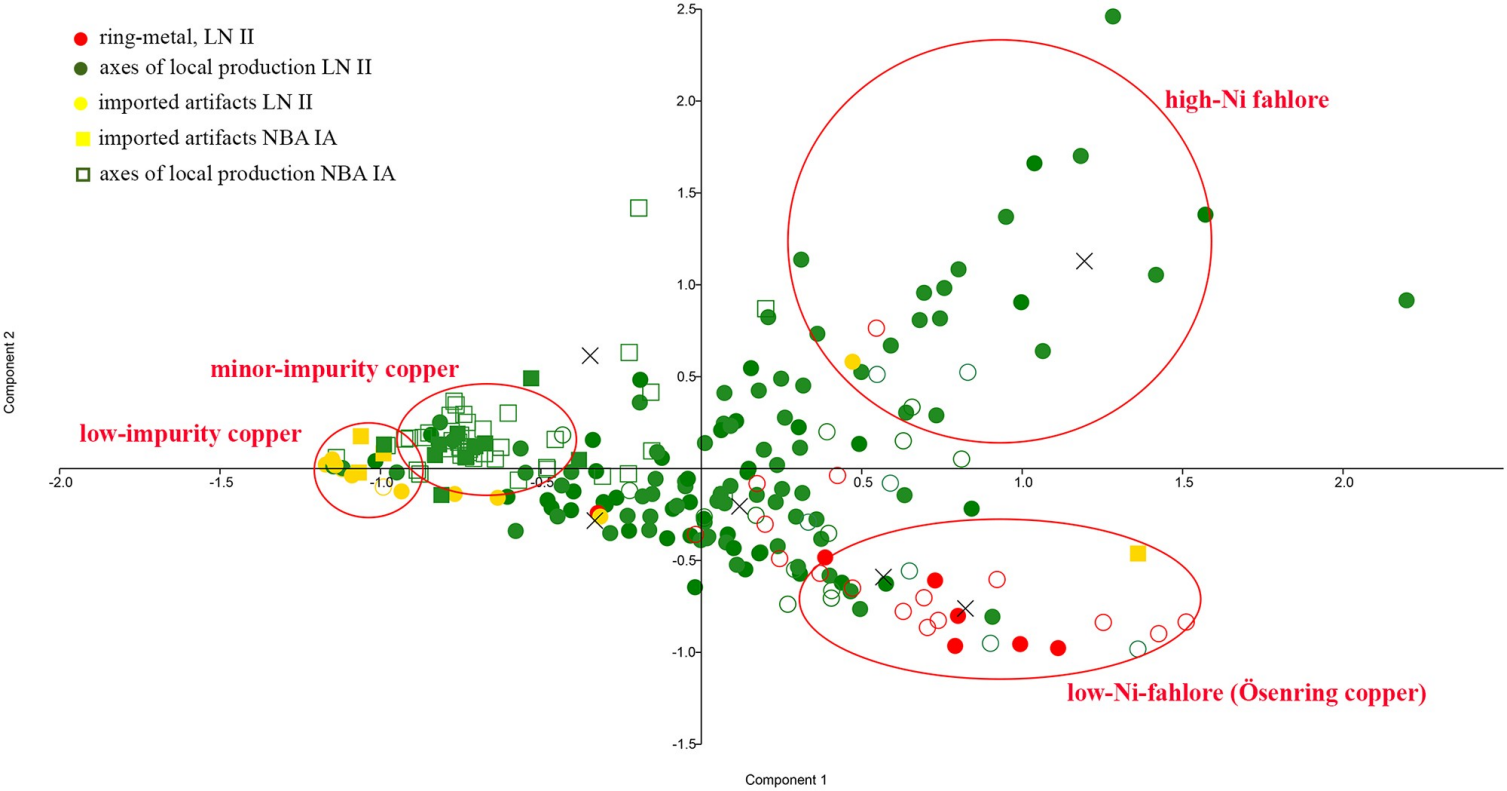
Principal Component Analysis of the main trace elements (Ni–Sb–Ag–As) of the LN II (dots) and NBA IA (squares) artifacts from Denmark, including the LN II Pile hoard in Scania. Generally, colors indicate the artifact category, the data set of Pile [19] is highlighted with open symbols. The extended data set is compared with the median values for copper types defined by Rassmann [91, 93], where Nieder–Neuendorf copper is Ni-free, copper types Bresinchen and Bennewitz have medium Ni values, and copper type Dederstedt has higher Ni values. Reinkupfer (pure copper) is defined, on the basis of several finds including the Helmsdorf burial, as very low-impurity copper [91].

A comparative Principal Component Analysis (cf. Fig 10) was therefore additionally run to clarify questions concerning the three definable fahlore types in the Danish material, investigating their association not only with each other, but with copper types in use in central Europe, here defined by Únĕtician hoards and burials in central Europe. These include high-Ni fahlore equal to Singen copper (Dederstedt), medium-Ni fahlore (Bresinchen), and non-Ni fahlore equal to *Ösenringkupfer* (Nieder–Neuendorf) [9, 65, 91, 93]. Indeed, the copper used by the Únĕtice culture reveals a comparable tripartite division of the early fahlore copper, which might indicate a provenance similar to the Danish equivalents. The high-Ni and medium-Ni fahlore groups in Denmark resemble Dederstedt, Bennewitz and, especially, Bresinchen copper in the Únĕtician hoards, while the Ni-free fahlore in Denmark and Scania very clearly recalls the Únĕtician Trebichau and Nieder–Neuendorf copper. This pattern of ‘similar but different’ is also found for several additional types of NBA IA artifact that cluster near the contemporaneous Kläden high-Ni fahlore type, without however being a complete match [19]. There are small but significant deviations between the Danish and Únĕtician copper groups during NBA IA that call for explanation.

**Fig 11:**
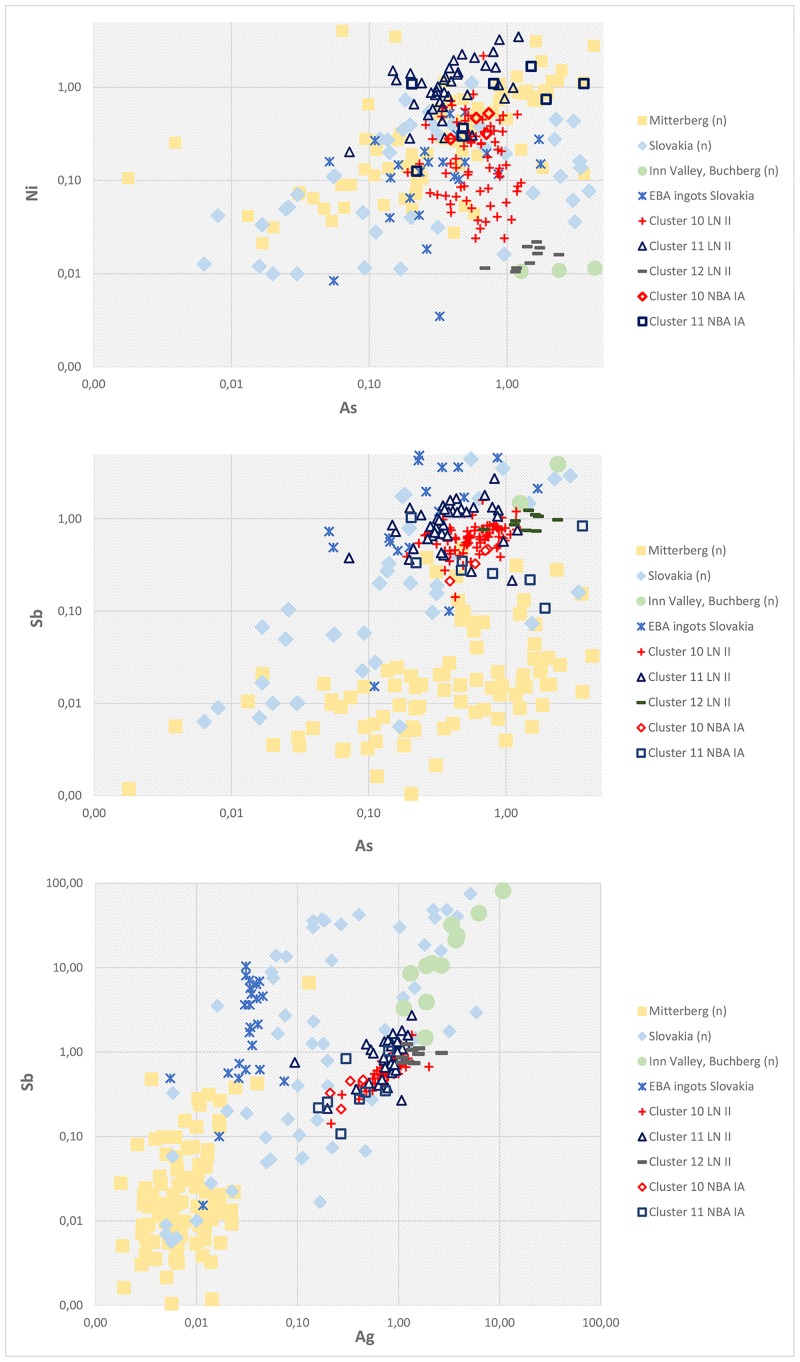
Trace element ratios of As–Ni, As–Sb and Ag–Sb of the fahlore ore copper groups compared to the relevant ore bodies (normalized values) in the Slovakian Ore Mountains and the eastern Alps. Ore values are normalized to copper and based on regional, interdisciplinary investigations of the specific mining regions [9, 59, 80, 81].

The Principal Component Analysis illustrates that the Ni-free *Ösenringkupfer* forms a distinct category in its own right, compared with the high- and medium-Ni fahlore groups. This demarcation recurs in other diagrams too (cf. Figs 4 and 11). From the Alps to Scandinavia, the Ni-free Ösenring copper was a primary copper, dissolving gradually through local processes of mixing in the course of Scandinavian LN II and central European Br A1–A2b/c. By NBA IA, c. 1700 BC, this primeval copper had apparently ceased to exist throughout temperate Europe. The Scanian Pile hoard provides the first strong evidence that *Ösenringkupfer* was used to craft local-style axes and that as early as 2000 BC this metal was being transported long-distance in the shape of neck-rings with rolled ends [19], while mixing with the two Ni-fahlore copper seems to have taken place. The artifacts analyzed within the current project suggest that the transformation of *Ösenringkupfer* into local-style axes in Denmark and Scania recurred throughout the LN II period; as seen in Fig 8, both ring-shaped artifacts and axes plot in similar ranges as the Ni-free Nieder–Neuendorf copper. This discovery corroborates Vandkilde’s recent result based on the Pile hoard that Ösenringe were treated as ingots to produce local artifact types [19] especially at the margins of the metal-using societies of the Bronze Age. This is further substantiated by the trace element patterns of Malchiner type daggers [94] and a lead isotope analysis on a similar dagger found in Sweden [21, 25].

Overall, these are clear indications of targeted mixing of similar-type fahlore copper in Scandinavia as well as in central Europe of the same period. It is possible that the mixing of two of the fahlore types–one with high Ni and the other Ni-free–could to some extent account for the third type of medium-Ni fahlore copper identified in and beyond Denmark. Since most of this fahlore type metal is isotopically consistent with the Slovakian Ore Mountains it is also possible that the variable Ni concentrations simply reflect the variability of the ore deposits. Nickel cannot be accommodated in the lattice of fahlore minerals and is present in other accessory minerals so that the nickel concentration can vary from ore charge to ore charge. If mixing has occurred then it may have been due to compensate for differences in mass among the newly produced objects, which would therefore require different amounts of metal. Regular mixing would have homogenized the chemical and lead isotope compositions, but such a distinct homogenizing was not observed. However, scattering of the lead isotope ratios among the locally crafted artifacts is so much lower than among the imported artifacts (Figs 8 and 9) that at least some of the locally crafted artifacts are likely to be the result of mixing. It may be worthwhile to note that in the case of mixing isotopically variable fahlore copper the so-called radiogenic isotope ratios with low values of e.g. ^206^Pb/^206^Pb tend to disappear as these low isotope values are always combined with very low lead concentrations. As for the British axes of LN II, these added an attractive golden color to the local products, and more specifically they increased the strength of the ‘natural fahlore alloys’ in this early period just preceding the implementation of conventional bronze technology. Furthermore, they generally contain more lead than the fahlore type copper. A related problem concerns the question whether certain forms were actually used as ingots. The mass-produced *Ösenringe* carrying the immensely uniform Ni-free copper–a prized commodity at the turn to the second millennium BC [65]–certainly could count as ingots, but undoubtedly in Scandinavia all metal objects were considered as potential ingots [19]. The literature is rich in discussions of this issue [90, 93]. Owing to a recognizable match between content and form, the diagram in Fig 8 even sustains the hypothesis that axe ingots and ring ingots represented fahlores with deviant properties, but the quite broad zone of compositional overlap here is a call for caution.

### Scandinavia’s early metallurgy: In pursuit of copper provenance—Central Europe

The targeted mixing of a limited number of different copper objects need not, however, erase all information on the provenance of Early Bronze Age copper [47, 90]. As 88% of the LN II artifacts were made of fahlore copper, it is appropriate to begin by focusing on provenancing this specific ore category. Between 2000 and 1700 BC, fahlore copper was known or mined in only a few regions–including Ross Island in southwest Ireland [88], which accommodated a less common type of fahlore consisting mainly of the arsenic sulfosalt tennantite with little antimony and easily distinguishable from central European fahlore type copper where the antimony sulfosalt tetrahedrite dominates. Other mining districts with a major output of fahlore minerals and attesting Bronze Age exploitation are the regions of Cabrières and Vallarde in southern France [95, 96], the Trentino in Italy [97], and the eastern Alpine region, including the Inn Valley [9, 48]. Fahlore deposits with secondary evidence of mining, such as mining tools, are also known in the Slovakian Ore Mountains [59, 80, 98]. In the Swiss Valais, lead isotope ratios suggested a connection between Bronze Age metal artifacts and local fahlore deposits [87, 99], though evidence for mining is meagre, consisting only of a few furnaces and slags related to Bronze Age sites [100]. Some of these regions also have accessory nickel minerals, and as the two major fahlore copper groups used in Nordic LN II have nickel as an impurity, those with low nickel concentrations such as Ross Island [88] and the Inn Valley in Tyrol [9, 81] can be excluded, apart from Schwaz-Brixlegg [101]. There are some minor traces of nickel in the Vallarde, and occasionally also in Cabrières [95], but because these mining regions flourished earlier, 3100–2500 BC [102], items produced at that time exhibit no stylistic connection to the artifact types of the Danish LN II and NBA IA (2000–1600 BC).

The lead isotope values of the three fahlore copper groups measured in the present project are consistent with the typochronological evidence and exclude the Trentino mining region with its significantly higher lead isotope values as a possible source (S2 Fig) [97, 103–105]. Exclusion of the Trentino therefore brings three main candidates to the fore: the Slovakian Ore Mountains and the Swiss Valais for the two Ni-containing fahlore copper groups (clusters 10–11), and the Inn Valley for the Ni-free fahlore copper of *Ösenring* affinity (cluster 12). The foregoing analysis has suggested that the source of the Ni-bearing fahlore should be sought in a different location from that with Ni-free fahlore. The typochronology and trace elemental patterns discussed above have significantly reduced the number of isotopically relevant ore bodies that are possible sources for the Scandinavian fahlore types. The Slovakian ore bodies [59, 80] are of significant interest for sourcing the fahlore copper, as are the Alpine region and specifically Buchberg in the Inn Valley [9, 81], in addition to the Valais region in western Switzerland [87]. Given the current state of research, however, Valais can only be assumed to be promising for future studies.

**Fig 12:**
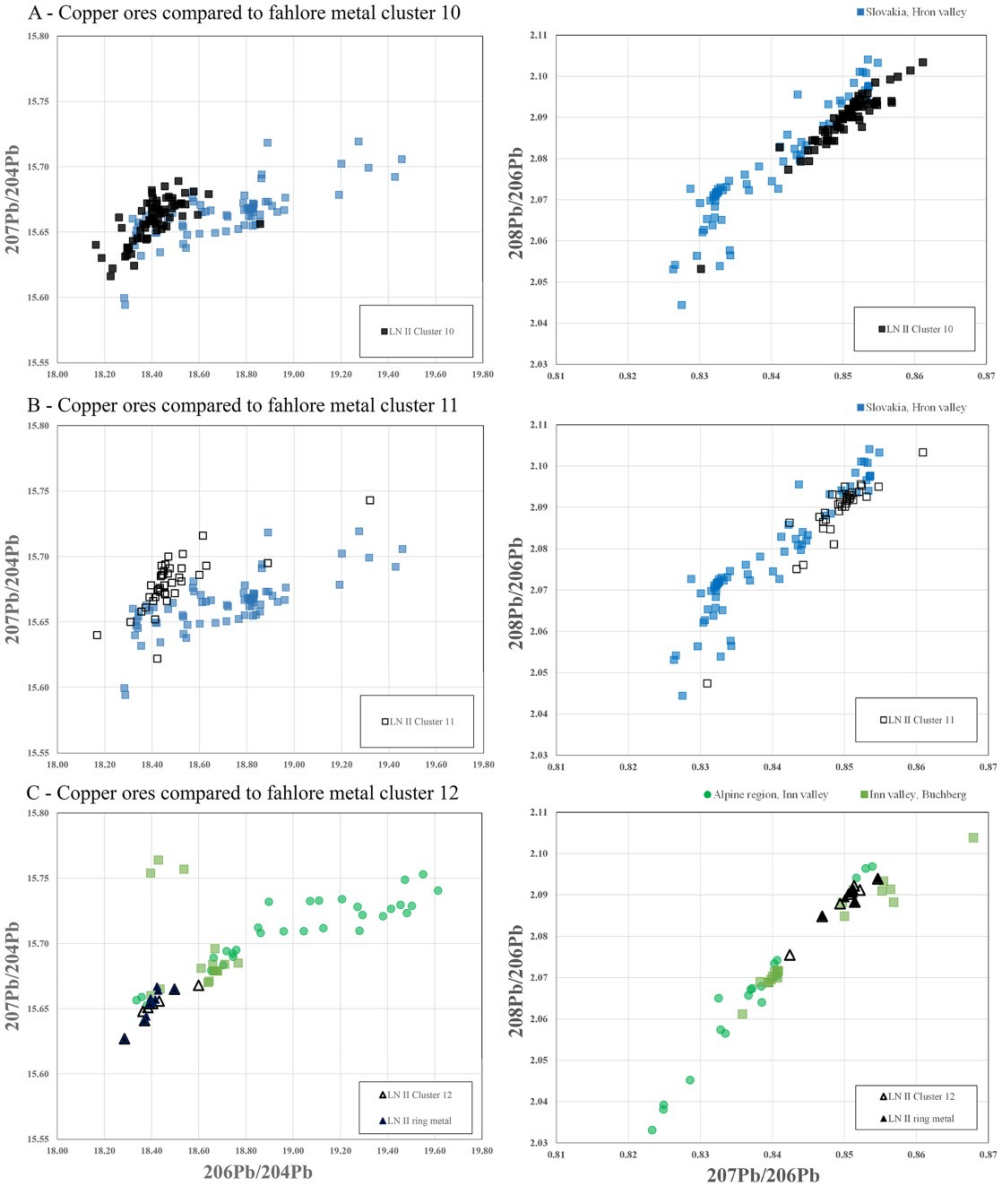
Lead isotope ratios of the different fahlore groups from LN II defined in this study compared with relevant ore regions. The plots clearly visualize the relation of (A-B) the Slovakian Ore Mountains with Ni-fahlore copper (the data represents the Hron valley, Slovakia [59, 80]), and of (C) the Inn Valley, especially the Buchberg, with Ni-free fahlore [9, 81].

Drawing on trace element data once again, As/Sb and Ag/Sb plots link the Scandinavian artifacts of Ni-fahlore metal to the Slovakian copper ores for both LN II and NBA IA. Furthermore, the trace element ratios of the Ni-free fahlore (cluster 12), especially the Ni–As ratios, strengthen standing assumptions that tie the *Ösenringkupfer* to the Inn Valley in the eastern Alps (Fig 11). The measured Ni-free samples from southern Scandinavia isotopically plot at similar values to indicative elements in ore samples from Buchberg in the Inn Valley, [81] very clearly pointing to an origin there (Fig 12). Remarkably, the rich ores at Mitterberg do not emerge as relevant at this point in time.

**Fig 13:**
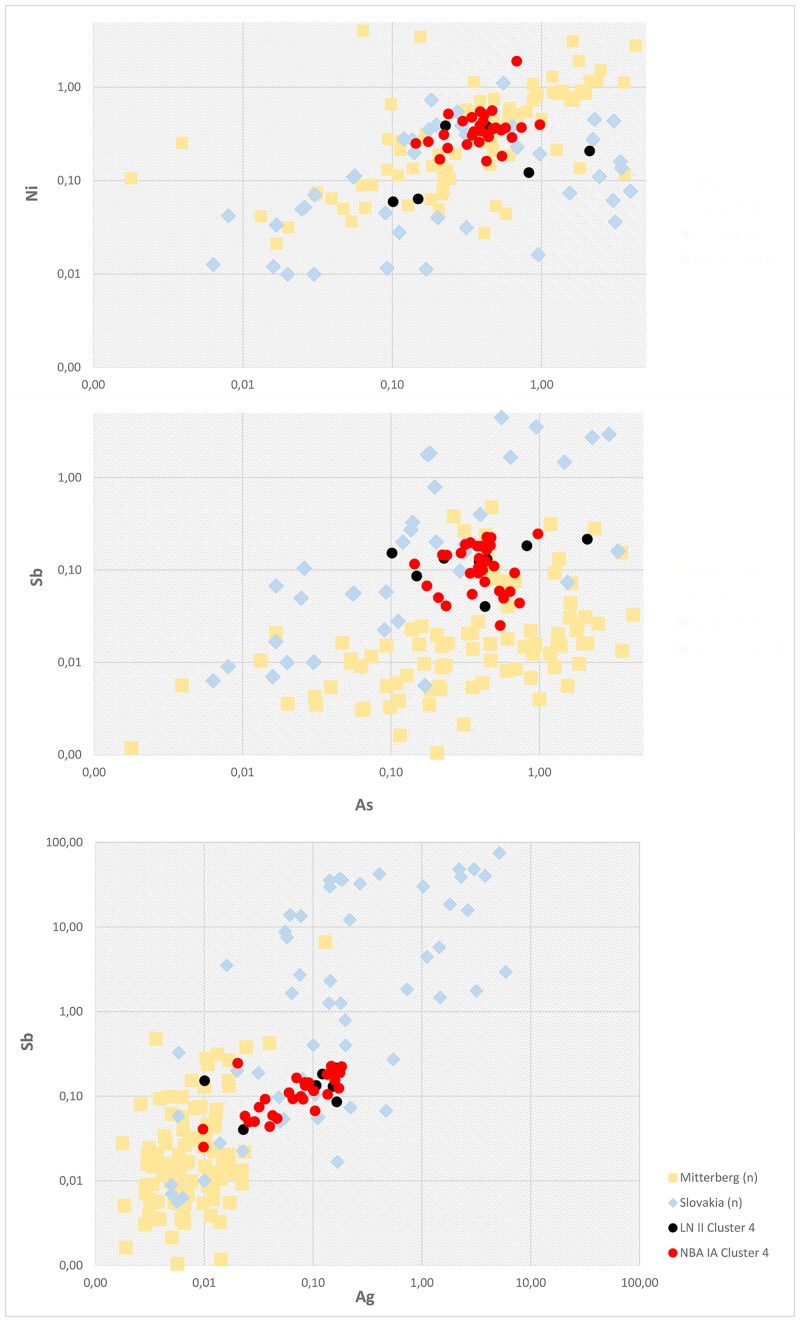
Logarithmic scatter plot of the trace elemental concentrations As–Ni, As–Sb and Ag–Sb of cluster 4 compared with the Mitterberg ore district and the Hron valley in the Slovakian Ore Mountains. Ore values are normalized to copper and based on regional interdisciplinary investigations of the specific mining regions [48, 59, 80].

Also noteworthy is the switch in copper compositions from LN II to NBA IA. This is clearly visible in the adoption of minor-impurity copper (alias cluster 4), which while only a tiny proportion of the copper used in LN II, it represents as much as 68% of the copper in NBA IA. The elemental composition of this copper group during NBA IA (Ni = As > Sb and absent/low Ag) is very similar to the characteristic trace element pattern of the greywacke zone of the eastern Alps and especially the Mitterberg region [48] with Ni = As (see Table 1). Critically, however, the lead isotope ratios fail to support Mitterberg as the provenance, since neither the ^206^Pb ratios nor the ^204^Pb ratios (Figs 6 and 9) display an undeniable similarity with copper ore samples from Mitterberg (though the southern district of the Mitterberg mining region, with its early mining activity, might still be a possibility as it is currently less well characterized in its range of lead isotope ratios). Nor are the Slovakian data from the Hron valley, including the mining regions at Banskà Štiavnica and Špania Dolina [59, 80], a perfect match with the ratios of this specific copper group (cluster 4) measured in the Scandinavian artifacts of LN II and NBA IA. Close inspection of the trace element patterns reveals that the Slovakian Ore Mountains are a more likely source for the NBA IA minor-impurity copper of cluster 4 –yet another pointer to Slovakia rather than the Mitterberg (Fig 13). An important point to make here is firstly that the minor-impurity cluster 4 could conceal copper from more than one mine or region and secondly that investigations concerning mining activity in the greywacke zone of Lower Austria are still ongoing. In addition, the transformation of imports into local forms by remelting and recasting may to a lesser degree have obscured information about geological provenance.

Inflows of British copper doubtless continue in NBA IA (Figs 5 and 9), nevertheless still with a distinct overweight of central European copper. This corresponds roughly to the LN II situation, also with regard to the Slovakian Ore Mountains as a major source area for the Scandinavian metal. Despite the new prominence of the minor-impurity copper of cluster 4, demand for Ni-fahlore copper continues throughout NBA IA. Its particular isotopic signals and trace compositions suggest that this latter copper was due to new arrivals rather than a continuous circulation of LN II copper. The minor-impurity (chalcopyrite) copper of NBA IA breaks with the ‘fahlore past’ and forecasts the ‘chalcopyrite future’ of the emerging Middle Bronze Age (Figs 5 to 9 and 11), as the analytical results of artifacts from 1600 BC onwards indicate [15, 45, 48].

### Scandinavia’s early metallurgy: In pursuit of copper provenance—The British Isles

**Fig 14:**
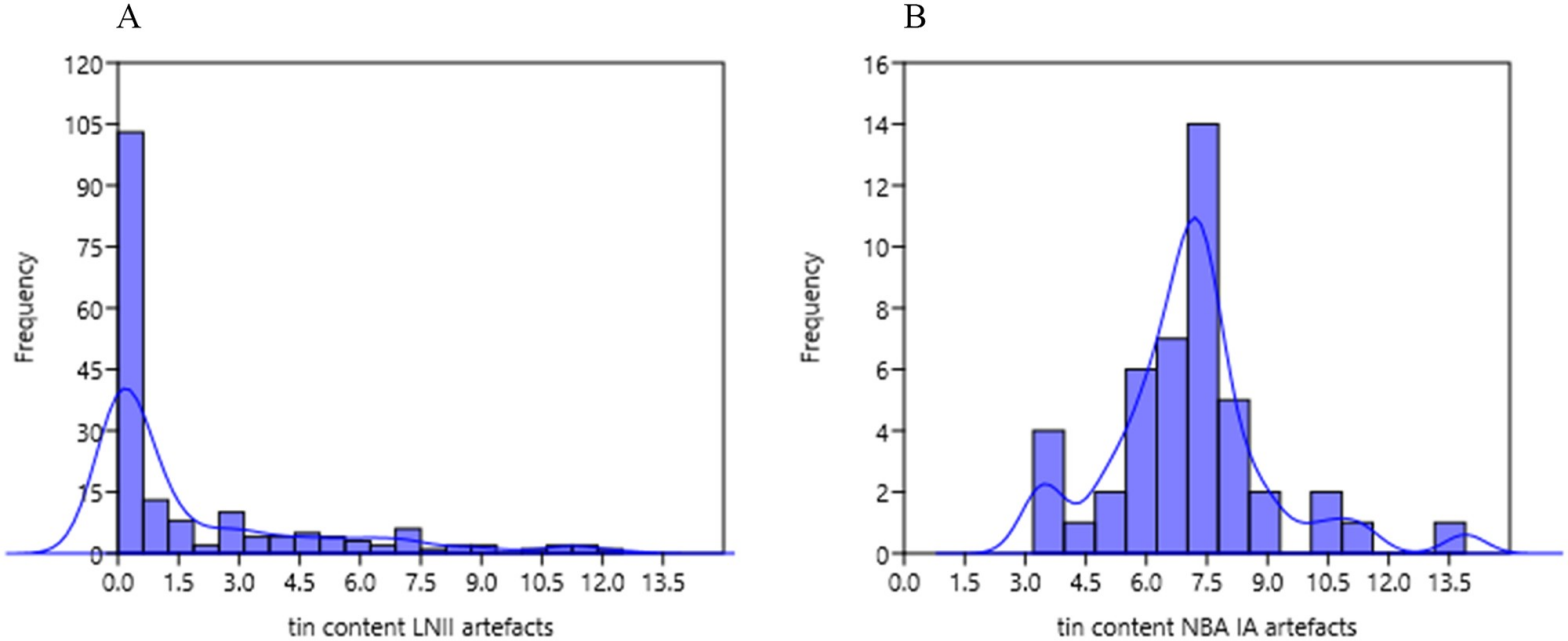
Histogram of the tin percentage within (A) LNII and (B) NBA IA artifacts found in Denmark.

**Fig 15:**
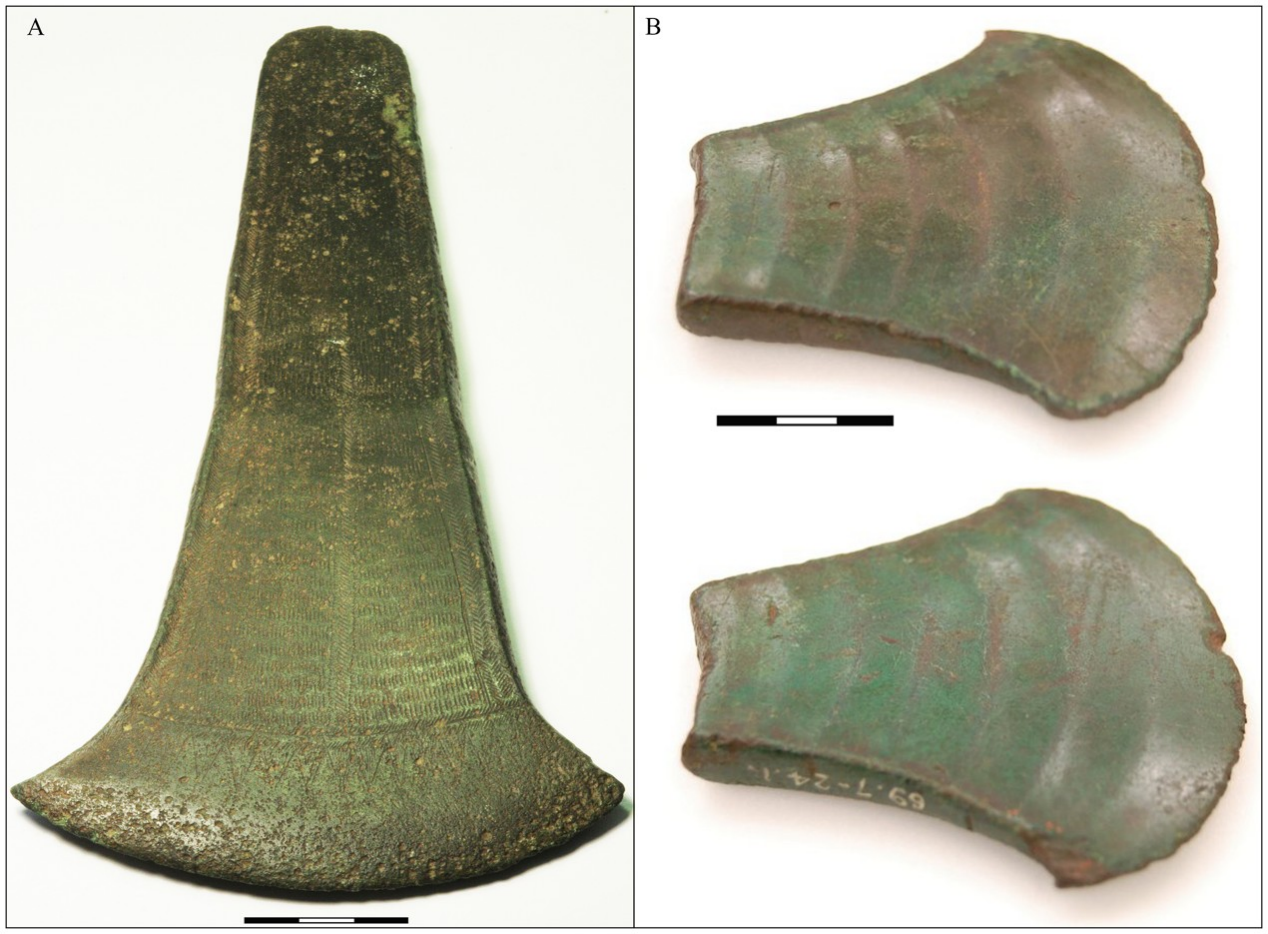
British axe metal in Denmark. (A) British-developed bronze flat-axe from Selchausdal, northwest Zealand (NM B5310, photo: Nørgaard). The 20-cm-long axe has a geometric decoration covering the surface. Low-impurity copper is alloyed with 10% Sn. Scandinavia holds the largest proportion of British type axes outside the British Isles 2000–1700 BC. (B) British axe interpreted as hack-metal found at Limfjord, Aalborg. Reprinted from [106] under a CC BY license, with permission from the Trustees of the British Museum.

Full implementation of the bronze alloy does not occur in Scandinavia until NBA IA when most local products have a tin content of 5–9%. This is in contrast to LN II, which has few high-tin bronze objects and mostly restricted to the group of British imports (Fig 14). A proportion of local LN II axes has Sn in the range 2–4% owing to tin added at some point in the life history of the copper. One likely explanation is the remelting of high-tin bronzework of central Únĕtician derivation (Erzgebirge tin?), but British high-tin bronze could very well be an additional source used to boost the beauty and strength of local products. The Scandinavian assortment of British high-tin axes [15, 49, 51] (Fig 15) specifically raises the question of the degree of, and reason for, the inclusion of British copper in the local production already during LN II, and how this source continues in use in NBA IA. One clue is the puzzling presence of western-style hybrids, including the pseudo-British axes (A8, see S1 Fig). Other clues are the low-impurity copper of clusters 1–3, 8–9, lead isotope signatures, and tin levels tending toward the higher end of the scale, indicating deliberate alloying in the original cast. It can be assumed that the tin derives from the rich Cornwall mines that account for the high tin value of British metalwork even prior to 2000 BC.

**Fig 16:**
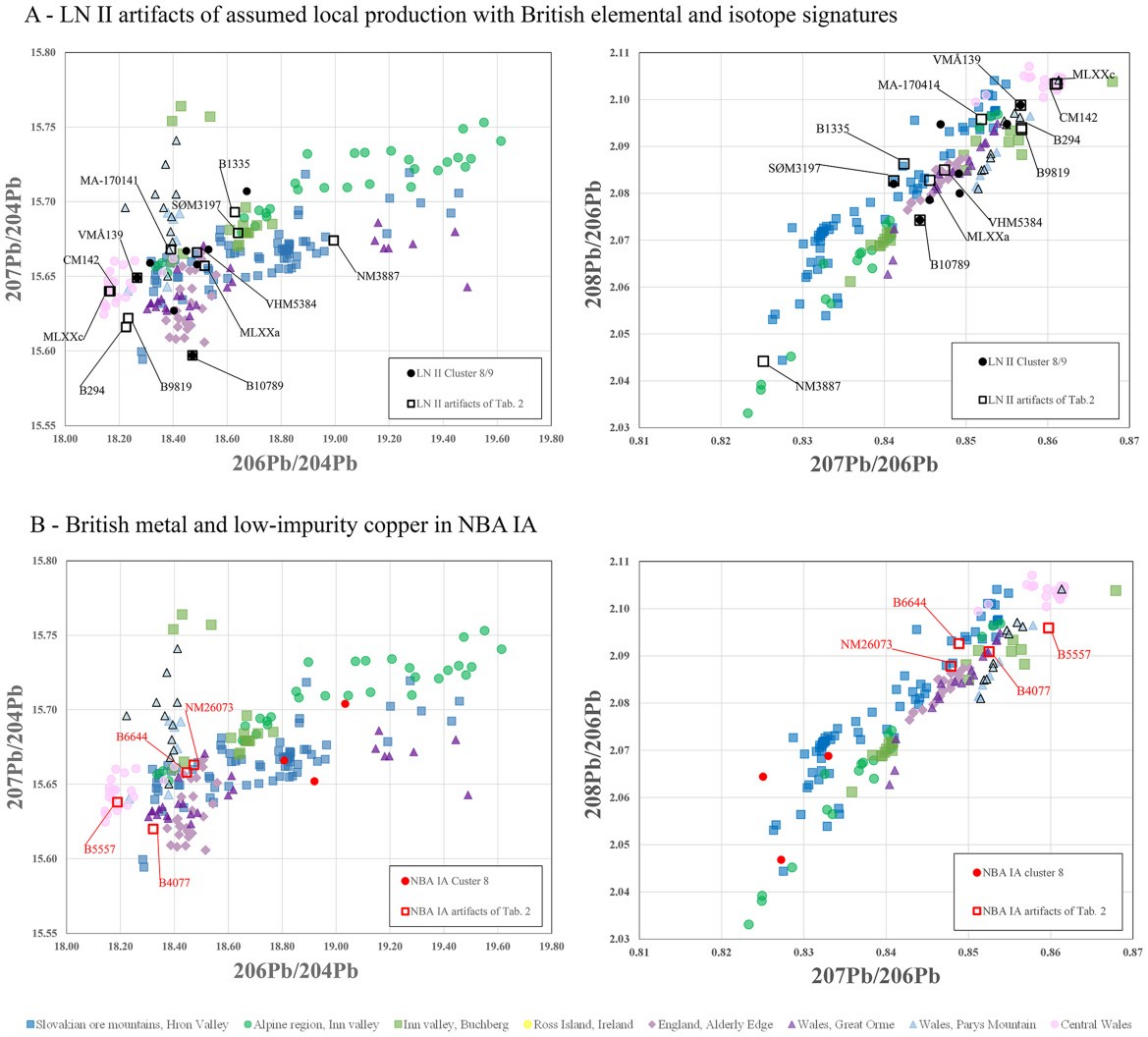
Lead isotope ratios of artifacts that can typologically be described as hybrids and locally crafted artifacts made of low-impurity copper (clusters 1, 8 and 9). (A) LN II artifacts with British lead isotope signatures (MLXX a, MLXXc, CM142, B294, B9819, B10789, VMÅ139 and VHM 5384) and local style artifacts (B1335, SØM 3197, NM3887) considered to be hybrids–(compare with Fig 8). (B) NBA IA artifacts that due to their isotopic signature are to be regarded as made (partly of) British high-tin metal (NM B644, B5557, B4077, NM26073). Conversely, the spread of cluster 8 artifacts suggests that the low-impurity copper used in the earliest Scandinavian Bronze Age derives from several different locations. The ore-related data are from: Ross Island [85, 86, 88]; Great Orme mining region, Wales [82–86]; north and central Wales mining regions [86, 89]; Alderley Edge mining region [85, 86]; eastern Alpine ores [9, 81]; Hron valley, Slovakian Ore Mountains [59, 80];.

With regard to LN II, the British axe in the Scanian Pile hoard is a case in point. Its tin content exceeds 11% and it has an atypical low-impurity composition for a continental European context, with isotopic values compatible with the Alderley Edge mine in Cheshire, UK [19, 90]. This piece was undoubtedly made in the British Isles, while a number of pseudo-British axes (A8) reveal remnants of British stylistic traits as well as British copper in their cast. A few distinctly local style axes also show a British signature in the composition of their copper. The analysis above has linked clusters 8–9 in particular to the British Isles, displaying high diversity lead isotope ratios with in ^206^Pb in the denominator and with ^204^Pb (Fig 8). Fig 14 exhibits the wide range of genuine British imports, hybrids, and a few local-style axes, predominantly with high tin content (Tables 2 and 3). An example is the pseudo-British hybrid with high tin from the Store Heddinge hoard (MLXXa, Fig 16) in east Zealand, which is fully compatible with British copper, perhaps from Alderley Edge or Great Orme. This axe can be assumed to have been recast in Denmark from a British original, as was probably also the case with several of the western-style hybrid axes (see Fig 16). A hybrid axe from the Odsherred hoard (B10789) [15] provides a good example of this, showing a distinct narrow waist recalling the Únӗtice axes but combined with British-style decoration. The axe from Dybvad Mølle, Jutland (VMÅ139) [15] similarly shows a stylistic blend, with middle-high tin (6%/ 8, 4%) on a low-impurity basis. The isotopic ratios support the assumption presented here (Table 2, Fig 16) and suggest that mines in northern Wales [86, 89] and Alderley Edge [107] respectively are possible sources for these two axes, while the medium tin values sustain the hypothesis of remelting. The exotic, large and spectacularly decorated British axes must also have been valued for their high tin contents, which provided color and strength in the local axes prior to the full adoption of bronze in NBA IA. On the other hand, a large axe with narrow waist and ‘British’ beveled decoration from Tolne Hjørring, in northern Jutland (NM3887) [15] still cannot be securely associated with British ores, perhaps indicating that cluster 8 brings together low-impurity copper from different sources (see Fig 16).

**Table 2:** Lead isotope ratios of artifacts of interest concerning the discussion of typological hybrids and foreign imports. The artifacts presented here are plotted in Fig 16.

sample MA	object	number	^208^Pb/^206^Pb	^207^Pb/^206^Pb	^206^Pb/^204^Pb	^208^Pb/^204^Pb Cal	^207^Pb/^204^Pb Cal	date
MA-171107	axe. type Gallemose	CM 142	21.033	0.86089	18.168	38.212	15.640	LN II
MA-171110	axe. type Gallemose	B294	20.936	0.85679	18.226	38.159	15.616	LN II
MA-171115	axe. type Gallemose	SØM3197	20.827	0.84113	18.641	38.823	15.679	LN II
MA-171127	axe. type Vaerslev	VMÅ139	20.988	0.85667	18.267	38.34	15.649	LN II
MA-170410	axe. type Store-Heddinge	MLXXc	21.034	0.86112	18.163	38.203	15.64	LN II
MA-166625	axe. type Store-Heddinge	B9819	20.940	0.85675	18.234	38.181	15.622	LN II
MA-170385	axe. type Aebelnaes	B10789	20.743	0.84435	18.472	38.318	15.597	LN II
MA-171122	axe. type Aebelnaes	VHM 5384	20.850	0.84734	18.489	38.549	15.666	LN II
MA-170408	axe Pseudo Anglo-Irish type	MLXXa	20.828	0.84555	18.517	38.567	15.657	LN II
MA-166635	axe. type Torsted-Tinsdahl	B1335	20.863	0.84238	18.629	38.865	15.693	LN II
MA-170414	axe. type Virring	NM	20.958	0.85186	18.392	38.547	15.668	LN II
MA-170358	axe	NM3887	20.442	0.82517	18.995	38.829	15.674	LN II
*MA-166638*	*axe*. *type Torsted-Tinsdahl*	*NM B6644*	*20*.*926*	*0*.*84885*	*18*.*446*	*38*.*601*	*15*.*658*	*NBA IA*
*MA-166648*	*axe*. *type Langenfeld*	*NM B5557*	*20*.*959*	*0*.*85974*	*18*.*189*	*38*.*123*	*15*.*638*	*NBA IA*
*MA-166656*	*axe*. *type Underaare*	*NM B4077*	*20*.*909*	*0*.*85251*	*18*.*322*	*38*.*308*	*15*.*620*	*NBA IA*
*MA-170418*	*axe*. *type Virring*	*NM26073*	*20*.*878*	*0*.*84787*	*18*.*473*	*38*.*568*	*15*.*663*	*NBA IA*

**Table 3:** Trace element values of the artifacts of interest concerning the discussion of typological hybrids and foreign imports. The artifacts presented here are related to Fig 16.

sample MA	object	number	cluster	Ni%	Sn%	As%	Ag%	Sb%	Pb%
MA-171107	axe. type Gallemose	CM 142	11	1.45	0.78	0.44	0.84	1.18	0.01
MA-171110	axe. type Gallemose	B294	10	0.14	2.49	0.43	0.83	0.62	0.06
MA-171115	axe. type Gallemose	SØM3197	10	0.07	0.83	0.31	0.69	0.53	0.00
MA-171127	axe. type Vaerslev	VMÅ139	8	0.03	8.5	0.10	0.01	0.01	0.04
MA-170410	axe. type Store-Heddinge	MLXXc	10	0.09	7.0	0.49	0.63	0.43	0.25
MA-166625	axe. type Store-Heddinge	B9819	10	0.15	0.10	0.23	0.90	0.54	0.02
MA-170385	axe. type Aebelnaes	B10789	9	0.02	6.0	0.01	0.01	0.00	0.00
MA-171122	axe. type Aebelnaes	VHM 5384	4	0.06	7.2	0.15	0.17	0.09	0.00
MA-170408	axe Pseudo Anglo-Irish type	MLXXa	4	0.08	11.3	0.08	0.23	0.19	0.01
MA-166635	axe. type Torsted-Tinsdahl	B1335	11	0.99	4.7	1.11	0.20	0.21	0.02
MA-170414	axe. type Virring	NM	4	0.33	6.6	0.44	0.15	0.13	0.10
MA-170358	axe	NM3887	4	0.35	6.1	0.39	0.10	0.12	0.01
*MA-166638*	*axe*. *type Torsted-Tinsdahl*	*NM B6644*	*4*	*0*.*40*	*10*.*8*	*0*.*98*	*0*.*02*	*0*.*25*	*0*.*15*
*MA-166648*	*axe*. *type Langenfeld*	*NM B5557*	*4*	*1*.*91*	*7*.*6*	*0*.*68*	*0*.*07*	*0*.*09*	*0*.*01*
*MA-166656*	*axe*. *type Underaare*	*NM B4077*	*4*	*0*.*37*	*8*.*1*	*0*.*57*	*0*.*03*	*0*.*05*	*0*.*40*
*MA-170418*	*axe*. *type Virring*	*NM26073*	*4*	*0*.*35*	*9*.*0*	*0*.*54*	*0*.*04*	*0*.*06*	*0*.*01*

**Fig 17:**
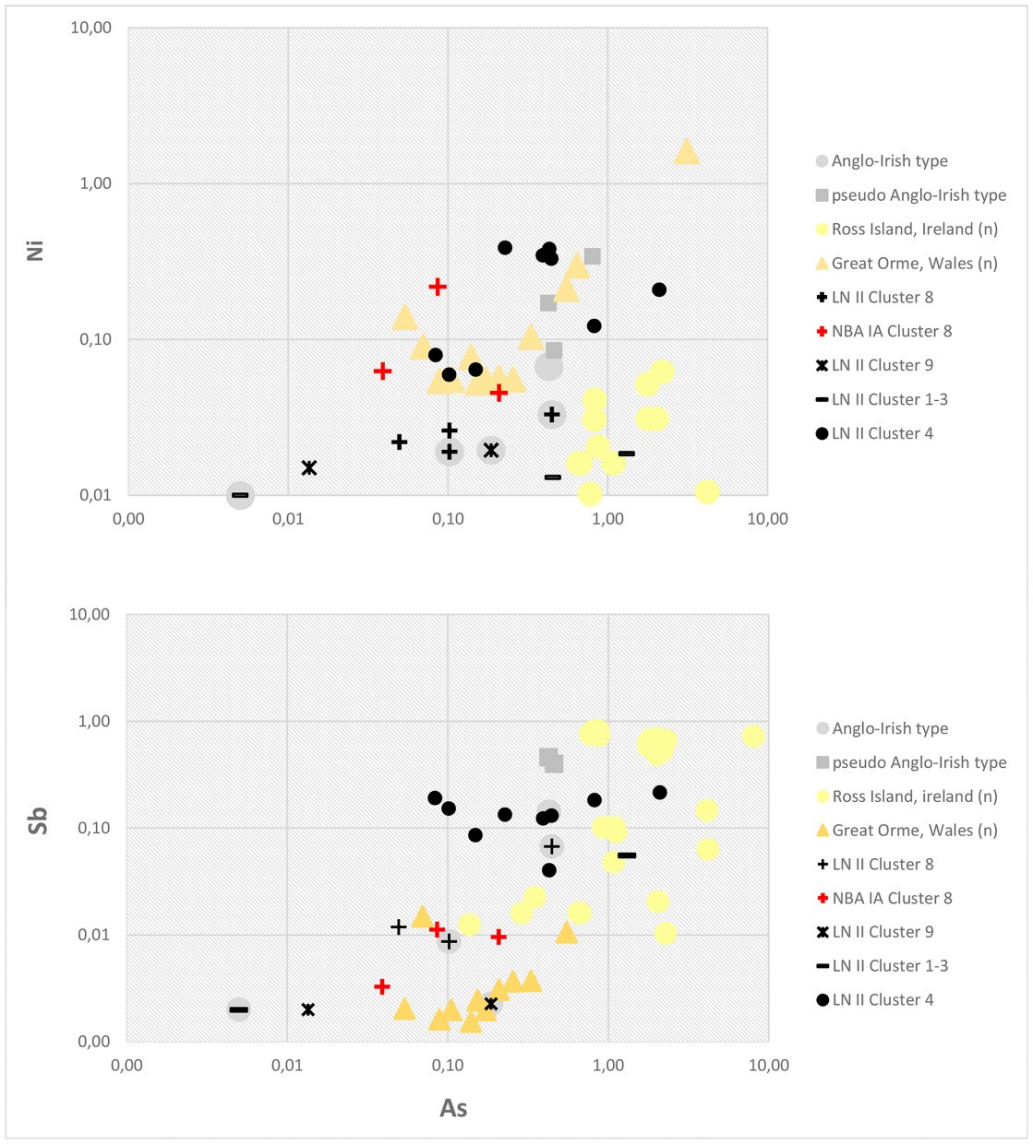
Trace element ratios (Ni/As, Sb/As) of the mining sites at Ross Island, Ireland, and Great Orme, Wales, compared with British and pseudo-British axes in addition to other artifacts in clusters 1–5, 8 and 9. Ore values are normalized to copper and based on regional interdisciplinary investigations of specific mining regions [82–86, 89].

Within the period 2000–1700 BC, mining activities in the British Isles were in a transitional phase. Production at the Ross Island mining site came to an end and several mines in Wales and England began to be exploited [82, 83, 88, 102, 108]. The broad spectrum of trace element composition measured in Scandinavian axes of probable British origin reflects this state of transition because they span four clusters of relatively pure copper. The Great Orme in Wales is a very good match for selected trace elements in these axes, while Ross Island in Ireland is not (Fig 17). Lead isotope data based on manifold data from these mining regions confirm the provenance from the British Isles [45, 82, 85, 86, 88, 89]. In how far mining sites in central and northern Wales, e.g. the Dyfed region as suggested by Melheim and others [45] or Parys Mountain [89], might be considered as potential sources is still a question to be addressed in future investigations. As for lead isotopes, Alderley Edge is clearly a possibility, as shown above, with at least two matches in addition to the British axe from Pile. The first use of the Great Orme mines is in fact contemporaneous with activities at the Alderley Edge mines [83, 107, 109–111]. Currently, however, there are no trace element data available for Alderley Edge nor from the contemporary mining sites in central and northern Wales, to strengthen these possibilities.

Turning to the succeeding period, **NBA IA**, the low-impurity copper of clusters 1–3 and 8–9 is still identifiable in this later dataset in similar proportions (e.g. Fig 5). The isotopic evidence of the few records, though, does not suffice to ascribe the low-impurity copper to the British mines without further discussion. As Fig 16 highlights, artifacts probably of British metal, predominantly deriving from Great Orme (Fig 16B), lie within cluster 4. Overall, NBA IA recalls the earlier situation that British metal is present. This likely represents a new supply of bronze to Scandinavia rather than the recycling of old metal from the same sources. The high isotopic diversity in the ^206^Pb ratios and ^204^Pb ratios in NBA IA matches the similarly wide range of British metal during LN II (Fig 8). Local metalwork and imports do not significantly diverge in this period in either style or the copper used.

The ratio of central European to British metal clearly favors the former both in LN II and in NBA IA, but it is nevertheless likely that British copper is underrepresented in the Scandinavian artifacts. The low-impurity hallmark of British copper (non-fahlore) would have meant the disappearance of British metal during repeated remelting and recasting more easily than central European copper with its high impurity levels–apart from high tin, which will remain visible in the data.

## Conclusions: Scandinavia’s early metallurgy in a wider Bronze Age world

Trading the local resource of amber enabled Scandinavia to enter the international marketplace in metals and metallurgy around 2000 BC by expanding in two directions (Fig 2). One route led down across the Baltic Sea toward the rich Únĕtice hubs in the Middle Elbe–Saale region. This connection stands out clearly in the archaeological data. In all likelihood it was maintained by frequent maritime and riverine travel, with the Mecklenburgian coastlands used as an easily accessible pick-up zone. The other route, also maritime but perhaps less routinely frequented, led to the British Isles, from where exotic axes were brought back to Scandinavia–indeed this Nordic collection boasts the largest number of British type axes outside the British Isles. These two major routes recur in the LN II data presented above, from minute typological details to micro-traces of the metals. Overall, analytical results bolster and nuance our current knowledge of Early Bronze Age Europe as tightly interconnected on scalar levels from micro to macro. New knowledge has emerged in the following areas:

Firstly, the transport of British axes to Scandinavia from England, possibly without the involvement of Ireland, figures much more clearly than previously. This indeed makes sense because of the relatively short distances involved when opting for routes along safe coastal waters (Fig 2). This result is visible in the combinations of data regarding elevated tin levels, low-impurity copper, and isotopic signatures in western-style axes. It also tallies with amber concentrations found mainly in the Wessex region [53, 54, 56, 112]. It is moreover possible that British bronze, and the western connection more broadly, is underestimated in the data.

Secondly, the geochemical characteristics of Scandinavian metal during LN II are strikingly similar to those found in the Únĕtice region. Minor but clear deviations between the two regions in elemental compositions can be accounted for by local mixing. The prevalent fahlore copper in all probability derived from the Inn Valley of the eastern Alps (Ni-free Ösenring copper) and from the Slovakian Ore Mountains (Ni copper groups). The lead role of Slovakia is unexpected and new and corroborates earlier studies in central Europe [113]. These two mining areas were vital players in the immense Únĕtician metal inventory. There is little in the archaeological data to suggest direct Scandinavian contact with the mining regions; rather, coveted metals reached Scandinavia through the agency of Únĕtice mediators, and in amounts that almost certainly exceeded shipments from Britain.

Thirdly, the investigations have thrown new light on the NBA IA period, the age that transitions to the final breakthrough of the Nordic Bronze Age c. 1600 BC. Though the seventeenth century BC is still shadowy, the NBA IA data patterns reveal both continuity and change compared to LN II. The same two-pronged trading connections, Britain and Únĕtice, continue to feed Scandinavian metallurgy. Local Nordic metalwork production on the one hand emerges as a cohesive autonomous tradition, yet on the other as clearly gravitating toward international standards of weaponry styles and techniques. Bronze is now fully implemented, as elsewhere in temperate Europe. Importantly, Ni-fahlore copper groups are still in use, but along with a new minor-impurity copper of cluster 4 (chalcopyrite) that has now rapidly become the prevailing copper type following its feeble introduction in LN II. Slovakia is still the lead provenance area, while the Inn Valley Ni-free fahlore has vanished. The Únĕtice region can probably still be attributed the role of mediator of Slovakian copper to Scandinavia up to c. 1600 BC, when the Únĕtician collapse paves the way for the emergence of the truly international Middle Bronze Age bringing Scandinavia into contact with the Mediterranean palace-based polities. The continued use of fahlore in NBA IA is reminiscent of the Earliest Bronze Age, while the prevalence of copper produced from ore that consist mainly of chalcopyrite forecasts new times. In the Middle Bronze Age, beginning around 1600 BC, chalcopyrite copper becomes utterly dominant, in many cases directly associated with industrial mining at Mitterberg in the eastern Alps.

Fourthly, the initial hypothesis of a local artifact production based on the smelting of raw copper and tin has been proven wrong. Compositional similarities between imports and local products in Scandinavia that imply interdependency hint strongly at the repeated remelting of imported artifacts (hacked into suitable pieces) as the direct source for local Nordic products, themselves also repeatedly recast. Since even the imports have a high variance in compositional patterns, the inference is plausible that these were also subject to mixing of the few primary copper types available as appropriate. This also suggests that ingots in the traditional meaning did not yet exist. Consistent with this is the evidence that the Bronze Age smith differentiated between axe metal (Ni fahlore) and ring metal (Ni-free fahlore) in dealing with two copper types with different properties. The British axes should be seen in the same light as quasi-ingots capable of providing tin. It is still unclear whether this system of mixing artifact metal, rather than properly alloying copper with tin, continued from LN II into NBA IA. To this can be added that early Scandinavian metal technology expresses individual characteristics at the same time as it relies on European role models.

## Supporting information

S1 Table Complete repository information of the artifacts used within this study. (PDF)

S2 Table Datasheet (Excel) of the lead isotope (MC-ICP-MS) and trace element (EDXRF) analyses executed on artifacts from LNII and NBA IA. (XLSX)

S1 Fig Grouping hierarchy of low-flanged axes of LNII [15]. The illustrations represent the most characteristic artifact of each type, the number below every artifact relates to the catalogue number in Vandkilde 1996.
(TIF)

S2 Fig Lead isotope ratios of the major European copper sources for the period 2100–1600 BC, which revealed primary or secondary traces of prehistoric activities. The ore data are from Mitterberg ore district [48]; Hron valley, Slovakian Ore Mountains [59, 80]; Inn Valley, Alpine region [9]; Buchberg, Inn Valley, Alpine region [81]; Trentino, Italy [97, 103–105], Ross Island [85, 86, 88]; north and central Wales mining regions [86, 89], Great Orme mining region, Wales [82–86]; Alderley Edge mining region [85, 86]; Valais valley, Switzerland [87]. The analytical uncertainties are comparable with the size of the symbols.
(TIF)
