# Correction: On the trail of Scandinavia’s early metallurgy: Provenance, transfer and mixing

**DOI:** 10.1371/journal.pone.0227504

**Published:** 2019-12-30

**Authors:** Heide W. Nørgaard, Ernst Pernicka, Helle Vandkilde

There is an error in Table 2. The data presented in the isotopic ratio column ^208^Pb/^206^Pb is incorrect due to misplaced decimal points. The authors have provided a corrected version here.

**Table 2:** Lead isotope ratios of artifacts of interest concerning the discussion of typological hybrids and foreign imports.

sample MA	object	number	^208^Pb/^206^Pb	^207^Pb/^206^Pb	^206^Pb/^204^Pb	^208^Pb/^204^Pb	^207^Pb/^204^Pb	date
MA-171107	axe. type Gallemose	CM 142	2.1033	0.86089	18.168	38.212	15.640	LN II
MA-171110	axe. type Gallemose	B294	2.0936	0.85679	18.226	38.159	15.616	LN II
MA-171115	axe. type Gallemose	SØM3197	2.0827	0.84113	18.641	38.823	15.679	LN II
MA-171127	axe. type Vaerslev	VMÅ139	2.0988	0.85667	18.267	38.34	15.649	LN II
MA-170410	axe. type Store-Heddinge	MLXXc	2.1034	0.86112	18.163	38.203	15.64	LN II
MA-166625	axe. type Store-Heddinge	B9819	2.0940	0.85675	18.234	38.181	15.622	LN II
MA-170385	axe. type Aebelnaes	B10789	2.0743	0.84435	18.472	38.318	15.597	LN II
MA-171122	axe. type Aebelnaes	VHM 5384	2.0850	0.84734	18.489	38.549	15.666	LN II
MA-170408	axe Pseudo Anglo-Irish type	MLXXa	2.0828	0.84555	18.517	38.567	15.657	LN II
MA-166635	axe. type Torsted-Tinsdahl	B1335	2.0863	0.84238	18.629	38.865	15.693	LN II
MA-170414	axe. type Virring	NM	2.0958	0.85186	18.392	38.547	15.668	LN II
MA-170358	axe	NM3887	2.0442	0.82517	18.995	38.829	15.674	LN II
*MA-166638*	*axe*. *type Torsted-Tinsdahl*	*NM B6644*	*2*.*0926*	*0*.*84885*	*18*.*446*	*38*.*601*	*15*.*658*	*NBA IA*
*MA-166648*	*axe*. *type Langenfeld*	*NM B5557*	*2*.*0959*	*0*.*85974*	*18*.*189*	*38*.*123*	*15*.*638*	*NBA IA*
*MA-166656*	*axe*. *type Underaare*	*NM B4077*	*2*.*0909*	*0*.*85251*	*18*.*322*	*38*.*308*	*15*.*620*	*NBA IA*
*MA-170418*	*axe*. *type Virring*	*NM26073*	*2*.*0878*	*0*.*84787*	*18*.*473*	*38*.*568*	*15*.*663*	*NBA IA*

The artifacts presented here are plotted in Fig 16.
