# Supplementary material for: On the trail of Scandinavia’s early metallurgy: Provenance, transfer and mixing
Source: PLoS One. 2019 Jul 24;14(7):e0219574. doi: 10.1371/journal.pone.0219574 (PMC6655661; doi:10.1371/journal.pone.0219574)
Supplement: S1 Table — (PDF) [file pone.0219574.s001.pdf]

Supplement 1: Alphabetical list of artifacts included in the present study with complete repository information.
* Detailed context information can be found in [15, 19, 26-44].

**Museum nb.**	**Museum collection**	**Object**	**Location**	**County**	**Region/ Denmark**	**Context***	**Date**	**artefact/ sample accessible**	**Sample number**
B949	Nationalmuseet København	flanged axe	Aebelnaes	Praestoe	Zealand	single find	LN II	yes	MA-171063
B949 (1)	Nationalmuseet København	flanged axe	Aebelnaes	Praestoe	Zealand	single find	LN II	yes	MA-171064
FHM 6095	Forhistorisk Museum Moesgaard	flanged axe	Albaek	Randers	Jutland	single find	LN II	yes	MA-166611
NM 1488	Nationalmuseet København	flanged axe	Als	Soenderborg	Jutland	single find	NBA IA	yes	MA-170433
B3484	Nationalmuseet København	flanged axe	Alsoenderup	Frederiksborg	Zealand	single find	LN II	yes	MA-170378
B8068	Nationalmuseet København	flanged axe	Amidgaard	Randers	Jutland	single find	LN II	yes	MA-171074
VMÅ139	Vesthimmerlands Museum	flanged axe	Dybvad Moelle (Ars)	Aalborg	Jutland	single find	LN II	yes	MA-171127
B2956	Nationalmuseet København	flanged axe	Balslev	Odense	Fyn	single find	LN II	yes	MA-170360
NM 26061	Nationalmuseet København	flanged axe	Barritskov	Vejle	Jutland	single find	LN II	yes	MA-170369
FSM 4009	Odense Bys Museer	flanged axe	Bogense	Odense	Fyn	single find	LN II	yes	MA-171106
B15117	Nationalmuseet København	spearhead	Bondesgaarde	Horsens	Jutland	multi-type hoard	NBA IA	yes	MA-180925
B15110	Nationalmuseet København	spearhead	Bondesgaarde	Horsens	Jutland	multi-type hoard	NBA IA	yes	MA-180926
B15115	Nationalmuseet København	spearhead	Bondesgaarde	Horsens	Jutland	multi-type hoard	NBA IA	yes	MA-180927
B15101	Nationalmuseet København	spearhead	Bondesgaarde	Horsens	Jutland	multi-type hoard	NBA IA	yes	MA-180928
B15131	Nationalmuseet København	spearhead	Bondesgaarde	Horsens	Jutland	multi-type hoard	NBA IA	yes	MA-180929
B15135	Nationalmuseet København	spearhead	Bondesgaarde	Horsens	Jutland	multi-type hoard	NBA IA	yes	MA-180930
FSM B294	Odense Bys Museer	flanged axe	Brandslund	Odense	Fyn	single find	LN II	yes	MA-171110
ML6574	Museum Lund	flanged axe	Breddinge	Schonen	Sweden	single find	LN II	yes	MA-181036
NM 26072	Nationalmuseet København	flanged axe	Brejl	Skanderborg	Jutland	single find	NBA IA	yes	MA-166640
NM 11073	Nationalmuseet København	flanged axe	Bygholm	Vejle	Jutland	single find	LN II	yes	MA-170370
FHM 3010	Forhistorisk Museum Moesgaard	flanged axe	Ebeltoft (near)	Randers	Jutland	single find	LN II	yes	MA-166616
FHM 5788	Forhistorisk Museum Moesgaard	flanged axe	Egtved	Vejle	Jutland	single find	LN II	yes	MA-166617
FHM 5554	Forhistorisk Museum Moesgaard	flanged axe	Egaa	Randers	Jutland	one-type hoard	LN II	yes	MA-166612
FHM 5554	Forhistorisk Museum Moesgaard	flanged axe	Egaa	Randers	Jutland	one-type hoard	LN II	yes	MA-166613
FHM 1179	Forhistorisk Museum Moesgaard	flanged axe	Egaa	Randers	Jutland	one-type hoard	LN II	yes	MA-166614
FHM 1179	Forhistorisk Museum Moesgaard	flanged axe	Egaa	Randers	Jutland	one-type hoard	LN II	yes	MA-166615
NM B6822	Nationalmuseet København	flanged axe	Ejstrup	Skanderborg	Jutland	single find	NBA IA	yes	MA-166641
B108	Nationalmuseet København	flanged axe	Ejstrup Holm	Skanderborg	Jutland	single find	NBA IA	yes	MA-170435
B8688	Nationalmuseet København	flanged axe	Bregnhoved	Vejle	Jutland	single find	LN II	yes	MA-170349
B13577	Nationalmuseet København	flanged axe	Ellesoe	Odense	Fyn	single find	LN II	yes	MA-170359
NM B3942	Nationalmuseet København	flanged axe	Emmerbølle	Svendborg	Fyn	single find	LN II	yes/no	MA-166633
FSM A395	Odense Bys Museer	flanged axe	Falsled	Svendborg	Fyn	single find	LN II	yes	MA-171100
NM 20828	Nationalmuseet København	flanged axe	Falster district	Maribo	Lolland	single find	LN II	yes	MA-166623
NM B13361	Nationalmuseet København	flanged axe	Fjellerup	Randers	Jutland	single find	NBA IA	yes	MA-166639
NM B13356	Nationalmuseet København	flanged axe	Flemloese	Odense	Fyn	single find	LN II	yes	MA-166635
FHM 26063	Forhistorisk Museum Moesgaard	flanged axe	Flenstofte	Odense	Fyn	single find	LN II	yes/no	MA-171084
ML6573	Museum Lund	flanged axe	Freddinge	Schonen	Sweden	single find	LN II	yes	MA-181035
NM 4415	Nationalmuseet København	flanged axe	Fyn	Fyn	Fyn	single find	NBA IA	yes	MA-166651
NM 15102	Nationalmuseet København	flanged axe	Fyn	Fyn	Fyn	single find	LN II	yes	MA-170352
FSM 27.6.1886	Odense Bys Museer	flanged axe	Faaborg	Svendborg	Fyn	single find	LN II	yes	MA-171102
B3887	Nationalmuseet København	flanged axe	Gallemose	Randers	Jutland	multi-type hoard	LN II	yes	MA-170389
B3888b	Nationalmuseet København	flanged axe	Gallemose	Randers	Jutland	multi-type hoard	LN II	yes	MA-170390
B3888c	Nationalmuseet København	flanged axe	Gallemose	Randers	Jutland	multi-type hoard	LN II	yes	MA-170391
B3888d	Nationalmuseet København	flanged axe	Gallemose	Randers	Jutland	multi-type hoard	LN II	yes	MA-170392
B3888e	Nationalmuseet København	flanged axe	Gallemose	Randers	Jutland	multi-type hoard	LN II	yes	MA-170393
B3888f	Nationalmuseet København	flanged axe	Gallemose	Randers	Jutland	multi-type hoard	LN II	yes	MA-170394
B3888g	Nationalmuseet København	flanged axe	Gallemose	Randers	Jutland	multi-type hoard	LN II	yes	MA-170395
B3889	Nationalmuseet København	ring	Gallemose	Randers	Jutland	multi-type hoard	LN II	yes	MA-170396
B3891a	Nationalmuseet København	open ring	Gallemose	Randers	Jutland	multi-type hoard	LN II	yes	MA-170397
B3891b	Nationalmuseet København	open ring	Gallemose	Randers	Jutland	multi-type hoard	LN II	yes	MA-170398
B3891c	Nationalmuseet København	open ring	Gallemose	Randers	Jutland	multi-type hoard	LN II	yes/no	MA-170399
B3891d	Nationalmuseet København	open ring	Gallemose	Randers	Jutland	multi-type hoard	LN II	yes	MA-170400
B3891e	Nationalmuseet København	open ring	Gallemose	Randers	Jutland	multi-type hoard	LN II	yes	MA-170401
B3891f	Nationalmuseet København	open ring	Gallemose	Randers	Jutland	multi-type hoard	LN II	yes	MA-170402
B3891g	Nationalmuseet København	open ring	Gallemose	Randers	Jutland	multi-type hoard	LN II	yes/no	MA-170403
B3885a	Nationalmuseet København	hook	Gallemose	Randers	Jutland	multi-type hoard	LN II	yes/no	MA-170404
B3885b	Nationalmuseet København	hook	Gallemose	Randers	Jutland	multi-type hoard	LN II	yes/no	MA-170405
B3886	Nationalmuseet København	hook	Gallemose	Randers	Jutland	multi-type hoard	LN II	yes/no	MA-170406
FSM 2649	Odense Bys Museer	flanged axe	Glavendrup	Odense	Fyn	single find	LN II	yes	MA-171104
FSM 2650	Odense Bys Museer	flanged axe	Glavendrup Mark	Odense	Fyn	single find	LN II	yes	MA-171099
B1043	Nationalmuseet København	flanged axe	Grinsted	Aalborg	Jutland	single find	LN II	yes	MA-170375
VHM 22391	Vedsyssel Historiske Museum	flanged axe	Guldagergaard	Hjoerring	Jutland	single find	LN I-II	yes	MA-171123
VHM 3208	Vedsyssel Historiske Museum	flanged axe	Gylling	Aarhus	Jutland	single find	LN II	yes	MA-171120
Thisted 1204	Museum Thy	flanged axe	Handrup Odde	Thisted	Jutland	single find	NBA IA	yes	MA-170438
B11333	Nationalmuseet København	flanged axe	Havdrup	Copenhagen	Zealand	single find	LN II	yes	MA-170383
NM 11011	Nationalmuseet København	flanged axe	Herslev	Holbaek	Zealand	single find	LN II	yes	MA-171078
NM 10183	Nationalmuseet København	flanged axe	Hjadstrup	Odense	Fyn	one-type hoard	NBA IA	yes	MA-166634
VHM 1945/78	Vedsyssel Historiske Museum	flanged axe	Hjoerring	Hjoerring	Jutland	single find	LN II	yes	MA-171121
FSM 8882	Odense Bys Museer	flanged axe	Holmdrup	Svendborg	Fyn	single find	LN II	yes	MA-171101
NM B300	Nationalmuseet København	flanged axe	Horbelev	Maribo	Lolland	single find	LN II	yes	MA-166627
ÅHM 3210b	Forhistorisk Museum Moesgaard	flanged axe	Hornumbro Moelle	Aalborg	Jutland	single find	LN II	yes	MA-171126

NM 10724	Nationalmuseet København	flanged axe	Hudevad	Odense	Fyn	single find	NBA IA	yes	MA-170416
Rud. B87	Langelands Museum, Rudkøbing	flanged axe	Humble	Svendborg	Fyn	single find	LN II	yes	MA-171095
B6469	Nationalmuseet København	halberd	Hvornum	Randers	Jutland	single find	LN II	yes	MA-171065
Maribo 770	Museum Lolland Falster	flanged axe	Idestrup	Maribo	Lolland	single find	LN II	yes	MA-171094
NM B8869	Nationalmuseet København	flanged axe	Jelling	Vejle	Jutland	single find	NBA IA	yes	MA-166649
Z99	Nationalmuseet København	flanged axe	Jerslev	Holbaek	Zealand	single find	LN II	yes	MA-170376
NM B5106	Nationalmuseet København	flanged axe	Jordloese	Holbaek	Zealand	one-type hoard	LN II	yes	MA-166629
NM B5106	Nationalmuseet København	flanged axe	Jordloese	Holbaek	Zealand	one-type hoard	LN II	yes	MA-166630
B13576	Nationalmuseet København	flanged axe	Jordloese	Holbaek	Zealand	single find	LN II	yes	MA-171085
Hol 16823	Museum Vestsjælland	flanged axe	Kalundborg	Holbaek	Zealand	single find	LN II	yes	MA-171087
AA896	Museum Sønderjylland Åbenrå	flanged axe	Kalvoe	Haderslev	Jutland	single find	LN II	yes	MA-171118
NM B9028	Nationalmuseet København	flanged axe	Katrinebjerg	Copenhagen	Zealand	single find	NBA IA	yes	MA-166645
B10900	Nationalmuseet København	flanged axe	Kildemosen	Frederiksborg	Zealand	single find	NBA IA	yes	MA-170441
FSM 496	Odense Bys Museer	flanged axe	Knorborg	Haderslev	Jutland	single find	LN II	yes	MA-171109
NM 7328	Nationalmuseet København	flanged axe	Korselitse	Maribo	Lolland	hoard?	LN II	yes	MA-170364
NM 21075	Nationalmuseet København	flanged axe	Kragebaek	Holbaek	Zealand	single find	NBA IA	yes/no	MA-166647
B10736	Nationalmuseet København	flanged axe	Kvaerkeby	Soroe	Zealand	single find	NBA IA	yes	MA-170417
FSM 4751	Odense Bys Museer	flanged axe	Kvaerndrupvaenge	Svendborg	Fyn	single find	LN II	yes	MA-171105
NM 1669	Nationalmuseet København	flanged axe	Langebjerg Mose	Odense	Fyn	single find	NBA IA	yes	MA-170430
B11203	Nationalmuseet København	halberd	Lejregaard	Copenhagen	Zealand	single find	LN II	yes/no	MA-171070
B1014	Nationalmuseet København	flanged axe	Lillerup	Skanderborg	Jutland	single find	LN II	yes	MA-170357
VSM 6121	Viborg Stiftsmuseum	flanged axe	Loevelbro Mark	Viborg	Jutland	single find	LN II	yes	MA-171128
NM 2730	Nationalmuseet København	flanged axe	Lumby Torp	Odense	Fyn	one-type hoard	LN II	yes	MA-170411
NM 2731	Nationalmuseet København	flanged axe	Lumby Torp	Odense	Fyn	one-type hoard	LN II	yes	MA-170412
NM 2732	Nationalmuseet København	flat axe	Lumby Torp	Odense	Fyn	one-type hoard	LN II	yes	MA-170413
ML11056	Museum Lund	flanged axe	Löddeköpinge	Schonen	Sweden	single find	LN II	yes	MA-181037
NM B9393	Nationalmuseet København	flanged axe	Magleby	Soroe	Zealand	single find	NBA IA	yes	MA-166646
NM 16201	Nationalmuseet København	flanged axe	Magleholm	Maribo	Lolland	single find	LN II	yes	MA-171073
SØM 4218	Odense Bys Museer	flanged axe	Mjang	Soenderborg	Jutland	single find	LN II	yes	MA-171113
NM B4077	Nationalmuseet København	flanged axe	Moskjaer	Randers	Jutland	single find	NBA IA	yes	MA-166656
NM B6655	Nationalmuseet København	flanged axe	Nakkehoved	Frederiksborg	Zealand	single find	LN II	yes	MA-166621
B9092	Nationalmuseet København	flanged axe	near Bogense	Odense	Fyn	single find	LN II	yes	MA-170372
B6678	Nationalmuseet København	flanged axe	near Davrup	Holbaek	Zealand	single find	LN II	yes	MA-170361
Maribo H25a	Museum Lolland Falster	flanged axe	near Maribo	Maribo	Lolland	single find	LN II	yes	MA-171092
Maribo H25b	Museum Lolland Falster	flanged axe	near Maribo	Maribo	Lolland	single find	LN II	yes	MA-171093
NM 2870	Nationalmuseet København	flanged axe	near Praesto	Praestoe	Zealand	single find	LN II	yes	MA-170384
NM 26073	Nationalmuseet København	flanged axe	near Silkeborg	Skanderborg	Jutland	single find	NBA IA	yes	MA-170418
NM B9390	Nationalmuseet København	flanged axe	near Skaelskoer	Soroe	Zealand	single find	NBA IA	yes	MA-166644
NM 26067	Nationalmuseet København	flanged axe	near Aalborg	Aalborg	Jutland	single find	LN II	yes	MA-171077
CM71	Odense Bys Museer	flanged axe	North Fyn	Odense	Fyn	single find	NBA IA	yes	MA-170428
HAD 795	Museum Sønderjylland Haderslev	flanged axe	Nustrup Parish	Haderslev	Jutland	single find	LN II	yes	MA-171112
NM B2162	Nationalmuseet København	flanged axe	Nysted	Maribo	Lolland	single find	NBA IA	yes	MA-166654
Horsens A4	Horsens Museum	flanged axe	Odder	Horsens	Jutland	single find	LN II	yes	MA-171119
B10790	Nationalmuseet København	flanged axe	Odsherred	Holbaek	Zealand	one-type hoard	LN II	yes	MA-170350
B10791	Nationalmuseet København	flanged axe	Odsherred	Holbaek	Zealand	one-type hoard	LN II	yes	MA-170381
B10789	Nationalmuseet København	flanged axe	Odsherred	Holbaek	Zealand	one-type hoard	LN II	yes	MA-170385
NM 20623	Nationalmuseet København	flanged axe	Pebertved	Svendborg	Fyn	single find	NBA IA	yes	MA-166643
B11127	Nationalmuseet København	flanged axe	Pederstrup	Maribo	Lolland	single find	LN II	yes	MA-170356
B1094	Nationalmuseet København	flanged axe	Pederstrup	Copenhagen	Zealand	single find	LN II	yes	MA-170377
B5118	Nationalmuseet København	halberd	Pederstrup	Copenhagen	Zealand	one-type hoard	LN II	yes	MA-171068
B5118	Nationalmuseet København	halberd	Pederstrup	Copenhagen	Zealand	one-type hoard	LN II	yes	MA-171069
NM 6838	Nationalmuseet København	flanged axe	Ramsoegaard	Copenhagen	Zealand	single find	LN II	yes	MA-170386
FSM B581	Odense Bys Museer	flanged axe	Refs-Vindinge	Svendborg	Fyn	single find	LN II	yes	MA-171098
NM B9819	Nationalmuseet København	flanged axe	Rye	Copenhagen	Zealand	single find	LN II	yes	MA-166625
NM B10993	Nationalmuseet København	flanged axe	Raabjerg	Hjoerring	Jutland	single find	NBA IA	yes	MA-166642
NM B3143	Nationalmuseet København	flanged axe	Saerslev	Holbaek	Zealand	single find	LN II	yes	MA-166632
NM 356	Nationalmuseet København	flanged axe	Saksgaard	Soenderborg	Jutland	single find	NBA IA	yes	MA-170432
B6676	Nationalmuseet København	halberd	Sandager	Svendborg	Fyn	single find	LN II	yes	MA-171066
FHM 2728	Forhistorisk Museum Moesgaard	flanged axe	Sejet	Skanderborg	Jutland	burial	LN II	yes	MA-171131
NM 4558	Nationalmuseet København	flanged axe	Selchausdal	Holbaek	Zealand	single find?	LN II	yes	MA-170353
B5310	Nationalmuseet København	flanged axe	Selchausdal	Holbaek	Zealand	single find?	LN II	yes	MA-170388
B1947	Nationalmuseet København	flanged axe	Sevel	Ringkoebing	Jutland	single find	NBA IA	yes	MA-181025
B98	Nationalmuseet København	flanged axe	Simmerboelle	Svendborg	Fyn	burial	NBA IA	yes	MA-170426
CM107	Odense Bys Museer	flanged axe	Skaerbaek Moelle	Haderslev	Jutland	single find	NBA IA	yes	MA-170429
B8687	Nationalmuseet København	flanged axe	Skannerup	Skanderborg	Jutland	single find	NBA IA	yes	MA-170422
NM 20948	Nationalmuseet København	flanged axe	Skenkelsoe	Frederiksborg	Zealand	single find	NBA IA	yes	MA-170421
FSM CM142	Odense Bys Museer	flanged axe	Skibby	Frederiksborg	Zealand	single find	LN II	yes	MA-171107
SØM 3198	Odense Bys Museer	flanged axe	Skodsboel	Soenderborg	Jutland	one-type hoard	LN II	yes	MA-171114
SØM 3197	Odense Bys Museer	flanged axe	Skodsboel	Soenderborg	Jutland	one-type hoard	LN II	yes	MA-171115
NM 11820	Nationalmuseet København	flanged axe	Skoerping	Aalborg	Jutland	single find	NBA IA	yes/no	MA-166653
B3240	Nationalmuseet København	flanged axe	Skovballe	Svendborg	Fyn	single find	LN II	yes	MA-170373
NM 9708	Nationalmuseet København	flanged axe	Skovsbogaard	Odense	Fyn	single find	NBA IA	yes	MA-170427
FHM 6299	Forhistorisk Museum Moesgaard	flanged axe	Sniderup	Viborg	Jutland	single find	LN II	yes	MA-171132
FHM 2994	Forhistorisk Museum Moesgaard	flat axe	Soerup Mark	Svendborg	Fyn	single find	NBA IA	yes	MA-171058
NM B9391	Nationalmuseet København	flanged axe	south Fyn	Svendborg	Fyn	single find	NBA IA	yes	MA-166636
Hol 9655	Museum Vestsjælland	flanged axe	Spaanbaekgaard	Holbaek	Zealand	single find	LN II	yes	MA-171090
B704	Nationalmuseet København	flanged axe	St. Duegaard	Bornholm	Bornholm	single find	LN II	yes	MA-170354
NM 16783	Nationalmuseet København	flanged axe	Stensby	Odense	Fyn	single find	NBA IA	yes	MA-166637

FHM 6264	Forhistorisk Museum Moesgaard	flanged axe	Stiling	Skanderborg	Jutland	single find	LN II	yes	MA-166619
NM B1517	Nationalmuseet København	flanged axe	Store Almegaard	Bornholm	Bornholm	burial	NBA IA	yes	MA-166652
NM MLXIX	Nationalmuseet København	flanged axe	Store-Heddinge	Praestoe	Zealand	one-type hoard	LN II	yes/no	MA-170407
NM MLXXa	Nationalmuseet København	flanged axe	Store-Heddinge	Praestoe	Zealand	one-type hoard	LN II	yes/no	MA-170408
NM MLXXc	Nationalmuseet København	flanged axe	Store-Heddinge	Praestoe	Zealand	one-type hoard	LN II	yes/no	MA-170410
B1182	Nationalmuseet København	flanged axe	Store-Toroeje	Praestoe	Zealand	one-type hoard	LN II	yes	MA-170365
NM B7534	Nationalmuseet København	flanged axe	Svendsmark	Praestoe	Zealand	single find	LN II	yes/no	MA-166622
B13980	Nationalmuseet København	flanged axe	Svinninge	Holbaek	Zealand	single find	LN II	yes	MA-170363
B6737	Nationalmuseet København	flanged axe	Svinsbjerg	Maribo	Lolland	single find	LN II	yes	MA-170355
NM B9392	Nationalmuseet København	flanged axe	Thise Hede	Viborg	Jutland	single find	NBA IA	yes	MA-166650
141/53	Roskilde Museum/ ROMU	flanged axe	Tjaereby	Copenhagen	Zealand	single find	NBA IA	yes	MA-170425
NM 3887	Nationalmuseet København	flanged axe	Tolne Parish	Hjoerring	Jutland	burial	LN II	yes	MA-170358
VHM 5384	Vedsyssel Historiske Museum	flanged axe	Tolne Parish	Hjoerring	Jutland	single find	LN II	yes	MA-171122
B10821	Nationalmuseet København	flanged axe	Tranderupgaard	Soroe	Zealand	single find	LN II	yes	MA-170382
NM B11334	Nationalmuseet København	flanged axe	Trustrup	Randers	Jutland	single find	LN II	yes	MA-166626
Horsens A2	Horsens Museum	flanged axe	Tvingstrup	Skanderborg	Jutland	single find	NBA IA	yes	MA-170436
B12993	Nationalmuseet København	flanged axe	Taarup	Maribo	Lolland	single find	LN II	yes	MA-170366
B6816	Nationalmuseet København	flanged axe	Ubberup	Holbaek	Zealand	single find	LN II	yes	MA-170380
FHM 140A	Forhistorisk Museum Moesgaard	flanged axe	Ulstrup	Viborg	Jutland	one-type hoard	LN II	yes	MA-166610
B3647	Nationalmuseet København	flanged axe	Vaerslev	Holbaek	Zealand	one-type hoard	LN II	yes	MA-171080
B3688	Nationalmuseet København	flanged axe	Vaerslev	Holbaek	Zealand	one-type hoard	LN II	yes	MA-171081
B3855	Nationalmuseet København	halberd	Veksoe	Frederiksborg	Zealand	single find	LN II	yes/no	MA-171071
S11243	Svendborg Museum	flanged axe	Vendsted Mose	Svendborg	Fyn	single find	LN II	yes	MA-171096
NM B5557	Nationalmuseet København	flanged axe	Vindebaekkobbel	Maribo	Lolland	single find	NBA IA	yes	MA-166648
NM 19198	Nationalmuseet København	flanged axe	Vindebæk	Praestoe	Zealand	single find	LN II	yes	MA-170362
B3962	Nationalmuseet København	flanged axe	Virring	Randers	Jutland	multi-type hoard	NBA IA	yes	MA-170423
B3963	Nationalmuseet København	flanged axe	Virring	Randers	Jutland	multi-type hoard	NBA IA	yes	MA-170424
B3958	Nationalmuseet København	spearhead	Virring	Randers	Jutland	multi-type hoard	NBA IA	yes	MA-180958
B3959	Nationalmuseet København	spearhead	Virring	Randers	Jutland	multi-type hoard	NBA IA	yes	MA-180959
B3960	Nationalmuseet København	spearhead	Virring	Randers	Jutland	multi-type hoard	NBA IA	yes	MA-180960
B3961	Nationalmuseet København	spearhead	Virring	Randers	Jutland	multi-type hoard	NBA IA	yes	MA-180961
NM B6644	Nationalmuseet København	flanged axe	Vorbasse	Ribe	Jutland	single find	NBA IA	yes	MA-166638
B10464	Nationalmuseet København	flanged axe	Vrold	Skanderborg	Jutland	burial	LN II	yes	MA-170367
Hobro 1001A	Naturhistorisk Museum Aalborg	flanged axe	West Himmerland	Aalborg	Jutland	single find	NBA IA	yes	MA-170440
B11683	Nationalmuseet København	flanged axe	West Lolland	Maribo	Lolland	single find	LN II	yes	MA-170371
NM 26062	Nationalmuseet København	flanged axe	Ørnboelle Hede	Hjoerring	Jutland	burial	LN II	yes	MA-170374
B7857	Nationalmuseet København	flanged axe	Ørum	Randers	Jutland	single find	LN II	yes	MA-170368
FSM CM104	Odense Bys Museer	flanged axe	Øster-Lunde	Odense	Fyn	single find	LN II	yes	MA-171103
B3494	Nationalmuseet København	flanged axe	Øster-Mellerup	Hjoerring	Jutland	single find	NBA IA	yes	MA-170415
AA901	Museum Sønderjylland Åbenrå	flanged axe	Aabenraa County	Aabenraa	Jutland	single find	LN II	yes	MA-171116
Ringk. 1710A	Ringkøbing-Skjern Museum	flanged axe	Aabjerg	Ringkoebing	Jutland	multi-type hoard	NBA IA	yes	MA-170437
NM B6821	Nationalmuseet København	flanged axe		Jutland	Jutland	single find	NBA IA	yes	MA-166655
B2430	Nationalmuseet København	flanged axe		Zealand	Zealand	single find	LN II	yes	MA-170379
NM 26060	Nationalmuseet København	flanged axe		Denmark	Denmark	single find	LN II	yes	MA-170387
NM NM	Nationalmuseet København	flanged axe		Denmark	Denmark	single find	LN II	yes	MA-170414
NM B4489	Nationalmuseet København	flanged axe		Denmark	Denmark	single find	NBA IA	yes/no	MA-170419
NM B13535	Nationalmuseet København	flanged axe		Denmark	Denmark	single find	NBA IA	yes/no	MA-170420
CM146	Odense Bys Museer	flanged axe		Zealand	Zealand	single find	NBA IA	yes	MA-170431
NM 2540	Nationalmuseet København	flanged axe		Denmark	Denmark	single find	NBA IA	yes	MA-170434
VHM 13835	Vedsyssel Historiske Museum	flanged axe		Denmark	Denmark	single find	NBA IA	yes	MA-170439
B4487	Nationalmuseet København	flanged axe		Denmark	Denmark	single find	LN II	yes	MA-171075
NM	Nationalmuseet København	flanged axe		Denmark	Denmark	single find	LN II	yes	MA-171076
DXCIX	Nationalmuseet København	flanged axe		Denmark	Denmark	single find	LN II	yes	MA-171082
B13536	Nationalmuseet København	flanged axe		Denmark	Denmark	single find	LN II	yes	MA-171083
B4486	Nationalmuseet København	flanged axe		Denmark	Denmark	single find	LN II	yes	MA-171086
Nt 1015/265	Næstved Museum	flanged axe		Denmark	Denmark	single find	LN II	yes	MA-171091
FSM CM99	Odense Bys Museer	flanged axe		Zealand	Zealand	single find	LN II	yes	MA-171108
FSM 4061	Odense Bys Museer	flanged axe		Denmark	Denmark	single find	LN II	yes	MA-171111
ÅHM 1578	Forhistorisk Museum Moesgaard	flanged axe		Denmark	Denmark	single find	LN II	yes	MA-171124
ÅHM 3384	Forhistorisk Museum Moesgaard	flanged axe		Denmark	Denmark	single find	LN II	yes	MA-171125
NM 1495	Nationalmuseet København	flanged axe		Denmark	Denmark	single find	LN II	yes	MA-171129
