# Supplementary figures and images for: On the trail of Scandinavia’s early metallurgy: Provenance, transfer and mixing
Source: PLoS One. 2019 Jul 24;14(7):e0219574. doi: 10.1371/journal.pone.0219574 (PMC6655661; doi:10.1371/journal.pone.0219574)

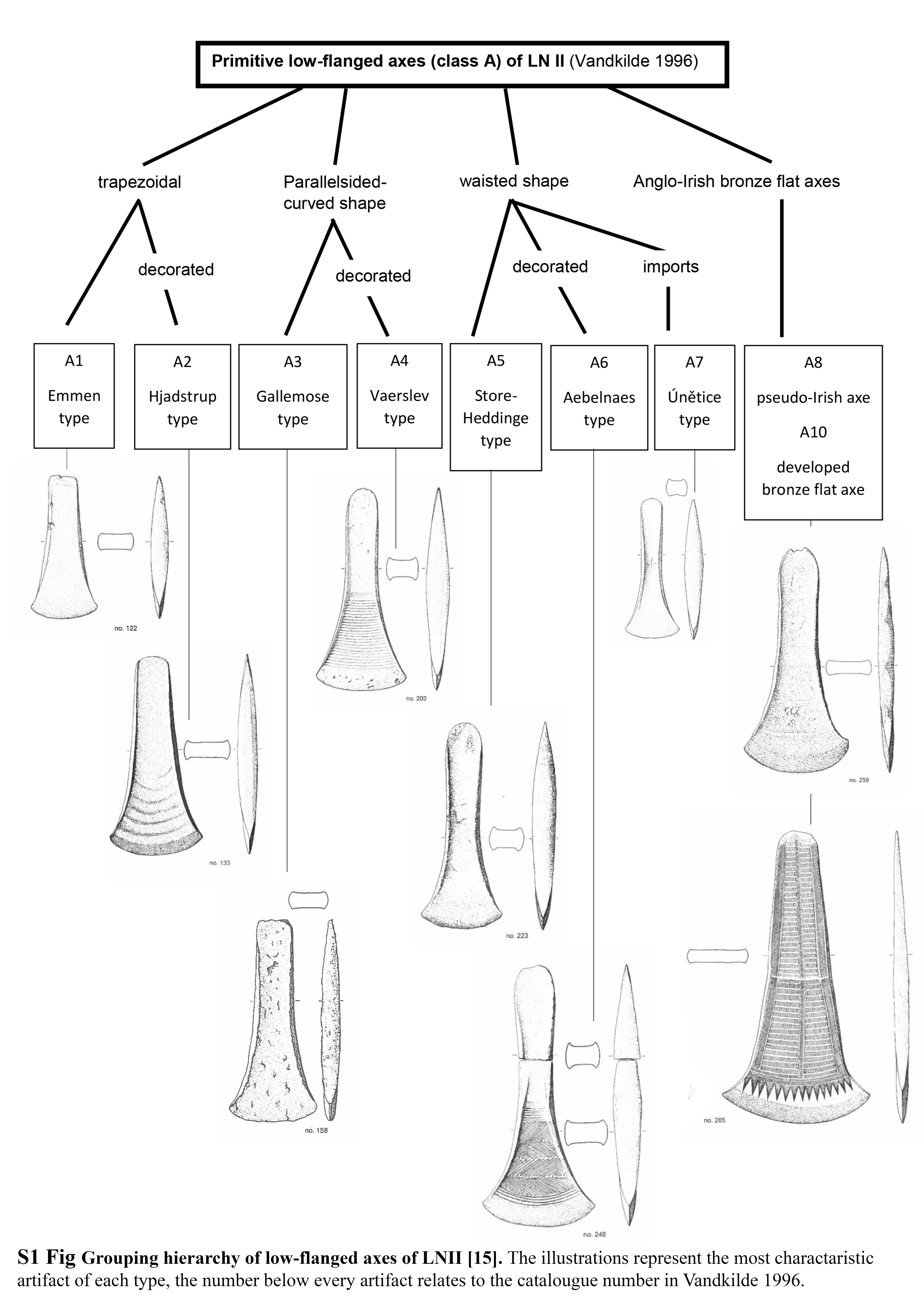

Supplement: S1 Fig — The illustrations represent the most characteristic artifact of each type, the number below every artifact relates to the catalogue number in Vandkilde 1996. (TIF) [file pone.0219574.s003.tif]

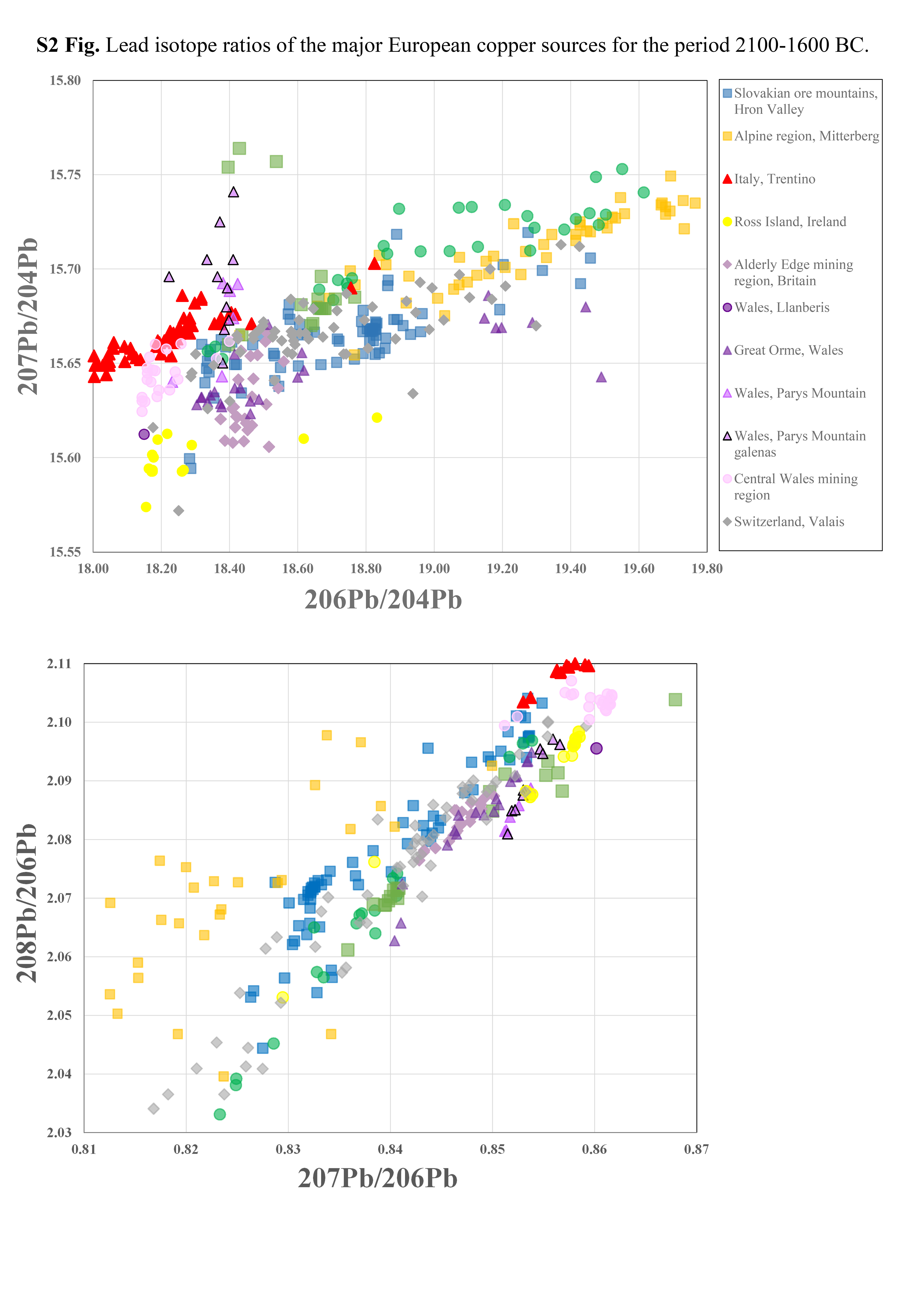

Supplement: S2 Fig — The ore data are from Mitterberg ore district [48]; Hron valley, Slovakian Ore Mountains [59, 80]; Inn Valley, Alpine region [9]; Buchberg, Inn Valley, Alpine region [81]; Trentino, Italy [97, 103–105], Ross Island [85, 86, 88]; north and central Wales mining regions [86, 89], Great Orme mining region, Wales [82–86]; Alderley Edge mining region [85, 86]; Valais valley, Switzerland [87]. The analytical uncertainties are comparable with the size of the symbols. (TIF) [file pone.0219574.s004.tif]
